# Molecular insights into the axon guidance molecules Sidestep and Beaten path

**DOI:** 10.3389/fphys.2022.1057413

**Published:** 2022-11-28

**Authors:** Caroline Heymann, Christine Paul, Na Huang, Jaqueline C. Kinold, Ann-Christin Dietrich, Hermann Aberle

**Affiliations:** ^1^ Department of Biology, Institute for Functional Cell Morphology, Heinrich Heine University Düsseldorf, Düsseldorf, Germany; ^2^ Institute of Neuro- and Behavioral Biology, University of Münster, Münster, Germany

**Keywords:** Drosophila, Sidestep, Beaten path, substrate attraction, axon guidance, neuromuscular junction

## Abstract

The transmembrane protein Sidestep (Side) functions as a substrate-bound attractant for motor axons in *Drosophila*. Outgrowing motor axons recognize Side *via* Beaten path Ia (Beat) and migrate along Side-expressing tissues. Here, we report a structure-function analysis of these guidance molecules using a variety of mutant lines and transgenic constructs. Investigation of Side mutants shows that the exchange of a single amino acid (L241H) in the second immunoglobulin domain disturbs Side function and subcellular localization. Overexpression of Side and Beat deletion constructs in S2 cells and muscles demonstrate that the first Ig domains of both proteins are necessary for their interaction. Furthermore, subcellular distributions of several Beat constructs identify functional domains and suggest a potential posttranslational processing step in ER compartments. In fact, fusing full-length Beat at both the N- and C-terminus with GFP and mCherry, respectively, shows that the N-terminal domain is transported to the plasma membrane and exposed on the cell surface, while the C-terminal domain accumulated in the nucleus. Taken together, these results give insights into the interaction of Side and Beat and imply that Beat might be subject to proteolytic cleavage during maturation.

## Introduction

The migration of axons during embryonic development is governed by attractive and repulsive guidance molecules that co-operate to control pathway selections over short or long distances. Based on the molecular nature of these cues, guidance decisions seem to be initiated in the extracellular space, either by transmembrane proteins or secreted factors. In the past decades, several protein families have been identified that are conserved across species and have related functions in vertebrates and invertebrates (Tessier-Lavigne and Goodman, 1996; Dickson, 2002; Kolodkin and Tessier-Lavigne, 2011).

Members of the Beaten path (Beat) and Sidestep (Side) families seem to be an exception from this principle, as they are not conserved in vertebrates or nematodes (Pipes et al., 2001; Vogel et al., 2003). However, highly homologous proteins are found in *Drosophila* and *Anopheles* species (Li et al., 2017). The founding members for each family, Beat and Side, were identified in unbiased screens for genes regulating motor axon guidance in *Drosophila* (van Vactor et al., 1993; Sink et al., 2001). Molecular cloning revealed that each protein classifies as a member of the immunoglobulin superfamily (Fambrough and Goodman, 1996; Sink et al., 2001). Beat consists of two N-terminal immunoglobulin (Ig) domains followed by a cysteine-rich region with similarities to cysteine knots (Bazan and Goodman, 1997; Mushegian, 1997). Side has five extracellular Ig domains followed by a potential fibronectin type-III domain, a transmembrane domain and a short cytoplasmic domain (Sink et al., 2001; Li et al., 2017). Although both proteins function in motor axon guidance and have similar mutant phenotypes, their expression pattern is quite different (Fambrough and Goodman, 1996; Sink et al., 2001). During embryogenesis, Beat is constantly expressed in motor neurons, but Side shows a spatiotemporally dynamic pattern that first emerges in glia cells (e.g. exit glia) of the CNS and then gradually changes to sensory neurons and muscles in the periphery. Functionally, Beat has been implicated in axon defasciculation at choice points, whereas Side was shown to attract motor axons. Besides motor nerves, Beat is also involved in development of the Bolwig’s nerve and its target, the Bolwig’s organ, which together constitute the larval visual system (Holmes and Heilig, 1999). Overexpression experiments in developing muscles confirmed that Beat has profound effects on the migration and defasciculation of axons (Fambrough and Goodman, 1996). Motor axons missed their targets or stalled at choice points. Such experiments were also pivotal for the identification of the attractive nature of Side. Misexpression of Side in trachea, in a wild-type background, caused motor axons to excessively explore tracheal branches (Sink et al., 2001). In a *side* mutant background, expression in hemocytes attracted many, if not most, motor axons to these highly motile cells, which are normally completely ignored by migrating axons (Siebert et al., 2009). Since Side is expressed along motor axon pathways it seems likely that it functions as a substrate label (Siebert et al., 2009).

Evidence for a functional interaction between Beat and Side is largely based on biochemical and genetic experiments. In cell culture, S2 cells singly transfected with either Beat or Side float in the medium but do not adhere to each other. When Beat- and Side-expressing cells are mixed, however, they aggregate in large cell-cell clusters (Siebert et al., 2009). Co-immunoprecipitations confirmed that Beat co-precipitates with Side and *vice versa* under these conditions (Siebert et al., 2009). Consistent with this, Beat and Side interact genetically, as double mutants have innervation phenotypes that resemble each single mutant (Siebert et al., 2009). Beat is also required to detect Side, because motor axons are no longer attracted to ectopic Side sources in *beat* mutant backgrounds (Siebert et al., 2009).

Recent large-scale interactome assays including most, if not all, members of the immunoglobulin superfamily in *Drosophila* confirmed direct Beat-Side interactions and, in addition, uncovered a network of interacting family members (Ozkan et al., 2013). An independent study “deorphanized” several more Beat-Side interactions (Li et al., 2017). The corresponding expression patterns indicate that Beats might primarily function as neuronal receptors, whereas Sides are more likely to represent directive cues at choice points or target cells (Li et al., 2017). It is hence conceivable that these proteins play a wider role in wiring the nervous system. Indeed, selective expression of particular Beats and Sides have been detected in defined cell types of the lamina in the fly visual system (Tan et al., 2015).

To gain further insight into the functional properties of these proteins, we generated a series of deletion constructs fused to fluorescent proteins and examined their subcellular distribution and protein-protein interactions in muscles. Deletion of the first Ig domain of Beat abrogated the association with Side, both in co-expression and co-immunoprecipitation experiments. Conversely, all Beat proteins containing the first Ig domain co-localized with exogenous Side. Moreover, deletion of the first Ig domain in Side failed to re-localize Beat-GFP, indicating that both proteins interact *via* their first Ig domains. Together with the finding that full-length Beat functions cell-autonomously in rescue experiments, these results further substantiate the idea that Beat is a membrane-tethered protein that detects Side in substrates.

## Materials and methods

### Genetics and fly stocks

Fly stocks were reared at 25°C on standard *Drosophila* cornmeal medium in plastic vials sealed with cellulose plugs. As control lines, *w* ^1118^ or ShGFP (also called CD8-GFP-Sh, insertion 7A on the III. chr.) were used (Zito et al., 1999). Point mutations in *beat* and *side* were isolated in unbiased EMS mutagenesis screens: *beat* ^*2*^, *beat* ^3^ (van Vactor et al., 1993), *side* ^D609^, *side* ^H143^ (Sink et al., 2001) and *sid*e^C137^, *side* ^I306^, *side* ^I1563^ and *side* ^K717^ (Aberle et al., 2002). The latter *side* alleles were induced in a ShGFP background. The inversion In(2L)TE146(Z)GR210^L^C163.41^R^ has a breakpoint in *beat*, here abbreviated as *beat* ^C163^ (Fambrough and Goodman, 1996). *Beat* ^2^ and *side* ^H143^ were kindly provided by David van Vactor (Harvard University, Cambridge, MA, United States), *beat* ^3^ (BL#4748) and *beat* ^C163^ (BL#4742) were ordered from the Bloomington *Drosophila* Stock Centre (BDSC, Indiana University, Bloomington, IN, United States). If not noted otherwise, transgenic fly lines were established by germline transformation using the site-specific ΦC31 integrase method (Bischof et al., 2007). Beat constructs were initially integrated at the recombination site Φ68E but later at Φ86Fa and Φ86Fb (all III. chr.) in attempts to improve low expression in the nervous system. All Side constructs were inserted at Φ51C (II. chr.). UAS-Beat-GFP^26.2^ was randomly integrated on the third chromosome using classical P-element transformation. UAS-Side^29A^ (Sink et al., 2001), UAS-Beat^5^ (Fambrough and Goodman, 1996), UAS-FasII and *side* ^D609^ were kind gifts of Corey Goodman (UC Berkeley, CA, United States). UAS-NSlmb-vhhGFP4 (BL#38421, BL#38422) was generously provided by Ulrich Thomas (IFN, Magdeburg, Germany) (Caussinus et al., 2011). Mef2-Gal4 has been described (Ranganayakulu et al., 1996) and Repo-Gal4 was obtained from BDSC (BL#7415). The motor neuron-specific driver FasII-Gal4^Mz507^ (B. Altenhein, University of Cologne, pers. comm.) was a valuable gift of Benjamin Altenhein (University of Cologne, Germany). Fat body-specific Pumpless (Ppl)-Gal4 and the hemocyte driver Serpent (Srp)-Gal4 were kindly supplied by Michael Pankratz (Limes Institute, Bonn, Germany) (Buch et al., 2008) and Rolf Reuter (University of Tübingen, Germany) (Huelsmann et al., 2006), respectively.

### DNA sequencing and sequence analysis

For sequencing *beat* and *side* alleles, genomic DNA was isolated from ∼100 heterozygous flies using the DNeasy Blood & Tissue Kit according to manufacturer instructions (Qiagen, Hilden, Germany). Exons adjacent intron sequences were amplified by PCR using intron-specific primers and proofreading Q5 High-Fidelity DNA Polymerase (New England Biolabs, Frankfurt a. Main, Germany). For determining the consequences of splice site mutations, total RNA was extracted from ∼20 homozygous larvae using the Totally RNA Kit following the manufacturer’s instructions (Life Technologies, Carlsbad, CA, United States). Total RNA was transcribed into cDNA using SuperScript III Reverse Transcriptase (Thermo Fisher Scientific, Schwerte, Germany). The mutated region was then amplified by PCR and sequenced (Eurofins Genomics, Ebersberg, Germany). For analysis, sequences were imported into MacVector Pro software (MacVector Incorporated, Apex, NC, United States) and aligned using T-Coffee.

### Molecular biology

Beat and Side constructs were designed by amplifying defined fragments of full-length *beat* and *side* cDNAs (Fambrough and Goodman, 1996; Sink et al., 2001) (kindly provided by Corey Goodman, UC Berkeley, CA, United States) by regular PCR or overlap extension PCR using proofreading polymerases. Full-length Beat contains the entire amino acid sequence but lacks a stop codon (aa 1–427). C-terminal deletions were named according to the encoded amino acids (e.g. Beat_1–322 comprises aa 1–322). For N-terminal deletions, BeatΔIg1 contains the endogenous signal peptide plus six adjacent amino acids (Beat aa 1–32) fused to Beat lacking the first Ig domain (aa 140–427). BeatCys comprises the endogenous signal peptide of Beat (aa 1–26) fused to the Beat C-terminus including the potential transmembrane domain (aa 320–427). Other Beat constructs modified potentially functional domains in Beat. BeatΔTM lacks a putative transmembrane domain (aa 324–343) and is composed of Beat aa 1–323 fused to aa 344–427. BeatnewTM contains Beat amino acids 1–323 fused to the transmembrane domain of human CD8 (IYIWAPLAGTCGVLLLSLVITLY) and the Beat Cys-rich domain (aa 344–427). Human CD8 sequences were derived from the vector pCasper MHC-CD8-GFP-Sh (Zito et al., 1999). GFP-Beat fuses the endogenous signal peptide (aa 1–26) to a short peptide linker (GSG), eGFP lacking a stop codon and Beat aa 27–427. Template for eGFP was the vector pUASTattB_rfA_eGFP (kindly provided by Floriano Rodrigues, Sven Bogdan and Christian Klämbt, University of Münster, Germany). Beat_29–427 replaces the endogenous signal peptide with that from human CD8 (aa 1–21), which is then fused to Beat aa 29 to 427. Beat_29–322 similarly contains the exogenous CD8 signal peptide fused to a C-terminally truncated form of Beat (aa 29–322).

Full-length Side contains the entire amino acid sequence without stop codon (aa 1–939). SideΔIg1 lacks the first Ig domain (aa 85–192) and fuses the endogenous N-terminus including the signal peptide (aa 1–84) in frame to truncated Side (aa 193–939). SideIg1-FasII-Cherry contains the Side Ig1 (aa 1–242) domain fused to Fasciclin II (FasII) lacking the first Ig domain (aa 142–873) using pBlueskript-FasII-PEST (Lin and Goodman, 1994) as template. To generate SideL241H-Cherry, total RNA of homozygous *side* ^*H143*^ embryos (sorted by hand) were isolated using NucleoSpin RNA Kit according to manufactures instructions (#740995, Macherey-Nagel, Düren, Germany). mRNA was transcribed into cDNA using SuperScript III Reverse Transcriptase (Thermo Fisher Scientific, Schwerte, Germany). The *side* cDNA containing the L241H point mutation was amplified by RT-PCR using proofreading Q5 High-Fidelity DNA Polymerase (New England Biolabs, Frankfurt a. Main, Germany) and forward and reverse primers 5′-CACC ATG​CAG​CTT​TTA​TTG​CCA​ACA​AGC and 5′-CTT​CAG​CGT​TGG​ATC​CAG​GGT​C, respectively.

All cDNAs were cloned into the Gateway Entry Vector pENTR D-Topo (Thermo Fisher Scientific, Schwerte, Germany) and sequenced from both ends. Full-length Beat was C-terminally fused to eGFP by *in vitro* recombination using LR clonase (Thermo Fisher Scientific, Schwerte, Germany) and the destination vector pTWG (11,473 bp, *Drosophila* Genomics Resource Center (DGRC), Bloomington, IN, United States; donated by Terence Murphy). All other constructs were C-terminally fused to either eGFP or mCherry (referred to GFP or Cherry throughout the manuscript) using the destination vectors pUASTattB_rfA_eGFP or pUASTattB_rfA_mCherry, respectively (kindly provided by Floriano Rodrigues, Sven Bogdan and Christian Klämbt, University of Münster, Germany). Due to the LR recombination sites, linker sequences encoding additional amino acids (KGGRADPAFLYKVVISAS/R) were inserted between cDNAs and C-terminal tags.

### Immunohistochemistry

Embryos were stained as described (Mahr and Aberle, 2006). Briefly, embryos were dechorionated, fixed with 3.7% formaldehyde and devitellinized. Embryos were washed with PTx (Phosphate-buffered saline (PBS) containing 0.1% Triton X-100), blocked in PTx/5% normal goat serum (NGS; Jackson ImmunoResearch, West Grove, PA, United States) and incubated with anti-Side antibodies on a nutator (VWR International, Leuven, Belgium) overnight at 4°C. Stainings were developed using fluorescently labeled secondary antibodies in PTx/5% NGS for 2h at room temperature. Embryos were washed in PTx and PBS, cleared in 70% glycerol/PBS and mounted on microscope slides (cover slips: 18 × 18 mm).

Third instar larvae (LIII) were dissected on PUCK dissection plates (Roland Vetter Laborbedarf OHG, Ammerbuch, Germany) using forceps (Dumont #5), microscissors (Vannas Spring Scissors, straight, 4 mm cutting edge) and 0.1 mm Minutien pins (equipment from Fine Science Tools, Heidelberg, Germany). Fillets were fixed in 3.7% formaldehyde, washed with PTx and blocked in PTx/5% NGS. After addition of primary antibodies, fillets were incubated overnight on a nutator at 4°C. Following three washing steps, fluorescently labeled secondary antibodies or Alexa568-Phalloidin were added in PTx/5% NGS and incubated for 2 h at room temperature on a nutator. Unbound antibodies were removed by three washes in PTx, and fillets were cleared in 70% Glycerol in PBS overnight. Elevated head and tail regions of the fillets were cut using microscissors, prior to mounting on microscope slides (cover slips: 22 × 22 mm).

Surface stainings were performed by dissecting LIII larvae in ice-cold PBS on PUCK dissection plates stored on crushed ice. The pinned and unfixed fillets were incubated with primary antibodies for 60 min on ice at a 10-fold higher concentration than for standard immunohistochemistry. Fillets were washed with ice-cold PBS, fixed with ice-cold 3.7% formaldehyde in PBS and rinsed with PTx at room temperature. Incubation with secondary antibodies, washing and mounting were performed as described above.

For standard immunohistochemistry, dilutions of primary antibodies were as follows: rabbit anti-GFP 1:1000 (TP401, Acris Antibodies, Herford, Germany), rabbit anti-dsRed 1:500 (#632496, TaKaRa, Mountain View, United States), mouse anti-Side 1:20 (clone 9B8; Developmental Studies Hybridoma Bank (DSHB), Iowa City, IA, United States), rabbit anti-GRP78 1:200 (NBP1-54318, Novus Biologicals, Centennial, United States) and mouse anti-Dlg 1:400 (clone 4F3; DSHB). Secondary antibodies (Jackson ImmunoResearch, West Grove, PA, United States) and Alexa568-Phalloidin (Thermo Fisher Scientific, Schwerte, Germany) were diluted 1:500 and 1:200, respectively.

### Immunoblotting

Overnight collections of control embryos containing all stages were dechorionated using standard procedures. For enrichment of stages 16–17, control embryos were dechorionated and manually separated under a stereo-microscope (Stemi 2000; Carl Zeiss MicroImaging, Jena, Germany). Homozygous mutant embryos were staged and manually sorted for loss of GFP-expressing balancer chromosomes using a UV stereo-microscope (M165 FC, Leica Microsystems, Wetzlar, Germany). Twenty embryos were lysed in 10 µl sample buffer (62.5 mM Tris/HCl, pH 6.8, 10 mM DTT, 2% (w/v) SDS, 10% (v/v) Glycerol, 0.02% Bromophenol blue) using a micropestle. Debris was removed by centrifugation for 5 min (14000rpm, RT). Supernatants were denatured at 98°C for 5 min before loading 20 μl in each slot of an SDS-PAGE gel.

Larval fillets were prepared as described above and homogenized in 25 µl sample buffer per fillet using a micropestle. After centrifugation (5 min, room temperature), the equivalent of one larval fillet was loaded in each slot of an SDS-PAGE gel. Samples were separated on 8% SDS-PAGE gels using Mini-PROTEAN Tetra Cells (Bio-Rad Laboratories, Feldkirchen, Germany) and transferred to PVDF membranes (Immun-Blot, Bio-Rad Laboratories, Feldkirchen, Germany) by semidry blotting using a Trans-Blot SD Semi-Dry Transfer Cell (Bio-Rad Laboratories, Feldkirchen, Germany). Blots were rinsed in TBST (Tris-buffered saline containing 1% Tween-20), blocked in TBST/5% powdered milk for 1 h at room temperature on a rocking platform shaker (VWR International, Leuven, Belgium). Blots were incubated with primary antibodies in blocking solution overnight at 4°C. The following primary antibodies were used: mouse anti-Side (1:100, clone 9B8, DSHB, Iowa, IA, United States), rabbit anti-GFP (1:5000; TP-401, Acris Antibodies, Herford, Germany), mouse anti-α-Tubulin (1:10,000; Sigma Aldrich, Taufkirchen, Germany). After washing in TBST, blots were incubated for 2 h at RT in blocking solution supplemented with horseradish peroxidase (HRP)-conjugated secondary antibodies (Jackson ImmunoResearch, West Grove, PA, United States) at a dilution of 1:7500. Membranes were washed in TBST and developed with WesternBright ECL HRP substrate (Advansta, San Jose, CA, United States). Molecular weights were standardized using prestained marker proteins (PageRuler; Thermo Fisher Scientific, Schwerte, Germany).

### Immunoprecipitation

Freshly prepared fillets of LIII larvae were homogenized using a micropestle in 15 µl lysis buffer (50 mM Tris/HCl, pH 7.5, 150 mM NaCl, 1% Nonidet P-40, Complete protease inhibitor [Roche Diagnostics, Mannheim, Germany]) per fillet, incubated on ice for 10 min and centrifuged (15 min, 14000rpm, 4°C). Supernatants derived from six fillets (90 μl) were collected and precleared for 1 h on an overhead test-tube rotator (Labinco, Breda, Netherlands) using protein A-sepharose beads (GE Healthcare, Uppsala, Sweden). Precleared lysates were incubated overnight with 5 µg rabbit anti-GFP antibodies (TP401, Acris Antibodies, Herford, Germany) on a test-tube rotator at 4°C. Protein A-sepharose beads (80 µl) were added, and tubes were rotated overhead for 3–4h at 4°C. Beads were collected by centrifugation (3 min, 3000g, 4°C) and washed 4x with 500 µl washing buffer (50 mM Tris/HCl, pH 7.5, 150 mM NaCl, 1% Nonidet P-40) at 4°C for 5–10 min on a nutator (VWR, International, Leuven, Belgium). Bound protein complexes were eluted with 60 µl sample buffer at 98°C for 5 min.

### Cell culture and aggregation assay

*Drosophila* S2 cells (R69007, Thermo Fisher Scientific, Schwerte, Germany) were grown at 28°C in complete Schneider’s *Drosophila* Medium (P04-90500, PAN-Biotech, Aidenbach, Germany), supplemented with 10% fetal bovine serum (P30-3302, PAN-Biotech, Aidenbach, Germany), 50 U/ml Penicillin/Streptomycin, 14.6 mg/ml Glutamine (10378016 Thermo Fisher Scientific, Schwerte, Germany). For routine passages, cultures were split 1:5 when they reached 90% confluence, generally every 3–4 days.

For cell aggregation assay, 2 ml S2 cells (1−3 × 10^6^) at the fifth to 10th passage were seeded into six-well plates on the day before transfection. Cells were transiently transfected using Effectene Transfection Reagent (301425, Qiagen, Hilden, Germany) and incubated at 28°C for 3 days. Average transfection efficiency was 9.5% for 500 ng of the GFP-Beat-Cherry construct. Transfected cells were seeded in a total volume of 600 μl into a round glass bottom dish (35 mm diameter; ibidi GmbH, Martinsried, Germany) and incubated at room temperature for 2 h with constant shaking at 100 rpm on a rocking platform (VWR International, Leuven, Belgium). Glass bottom dishes were examined for cell aggregates using confocal microscopy.

### Microscopy

Microscopic images were recorded using an inverted confocal laser scanning microscope (LSM710, Carl Zeiss MicroImaging, Jena, Germany). Raw images (1024 × 1024 pixel, line averaging 2) were processed using Fiji is just ImageJ software (Schindelin et al., 2012). Other pixel dimensions were either tile scans or image details. Z-stacks were compressed as maximum intensity projections in all figures. Figures were assembled using Adobe Illustrator and Adobe Photoshop (Adobe Systems, Dublin, Republic of Ireland). Anterior is left and dorsal is up in embryonic and larval images.

Living LIII larvae were picked at the wandering stage, immobilized by immersion into a 60 °C water bath for 1 s and mounted onto microscope slides in 70% Glycerol/PBS. Oriented larvae were covered with a 22 × 22 mm cover slip and immediately imaged for up to 30 min using 20x air/NA 0.8 or 63x oil/NA1.4 objectives (Plan Apochromat, Carl Zeiss MicroImaging, Jena, Germany). These objectives were also employed for imaging fixed and stained fillets preparations.

### Statistical analysis

Statistical diagram in Figure 3L displays the number of NMJs on dorsal-most muscles 1/9 and 2/10 (mean, standard deviation). Data sets were tested for normal distribution using the Kolmogorov-Smirnov test with Lilliefors correction. *p*-values were determined using two-tailed Mann-Whitney *U* test for not normal distributed data sets using Microsoft Exel (***p ≤ 0.01). For quantitative evaluation of the S2 cell-cell interaction assays, 30 aggregates were randomly selected, and their surface area was determined using the “measure” function in Fiji is just ImageJ software. *p*-values were determined by two-tailed Mann-Whitney *U* test. ns, non-significant, ***p ≤ 0.001.

## Results

### Sequencing of *side* alleles reveals EMS-induced missense and nonsense mutations

**FIGURE 1:**
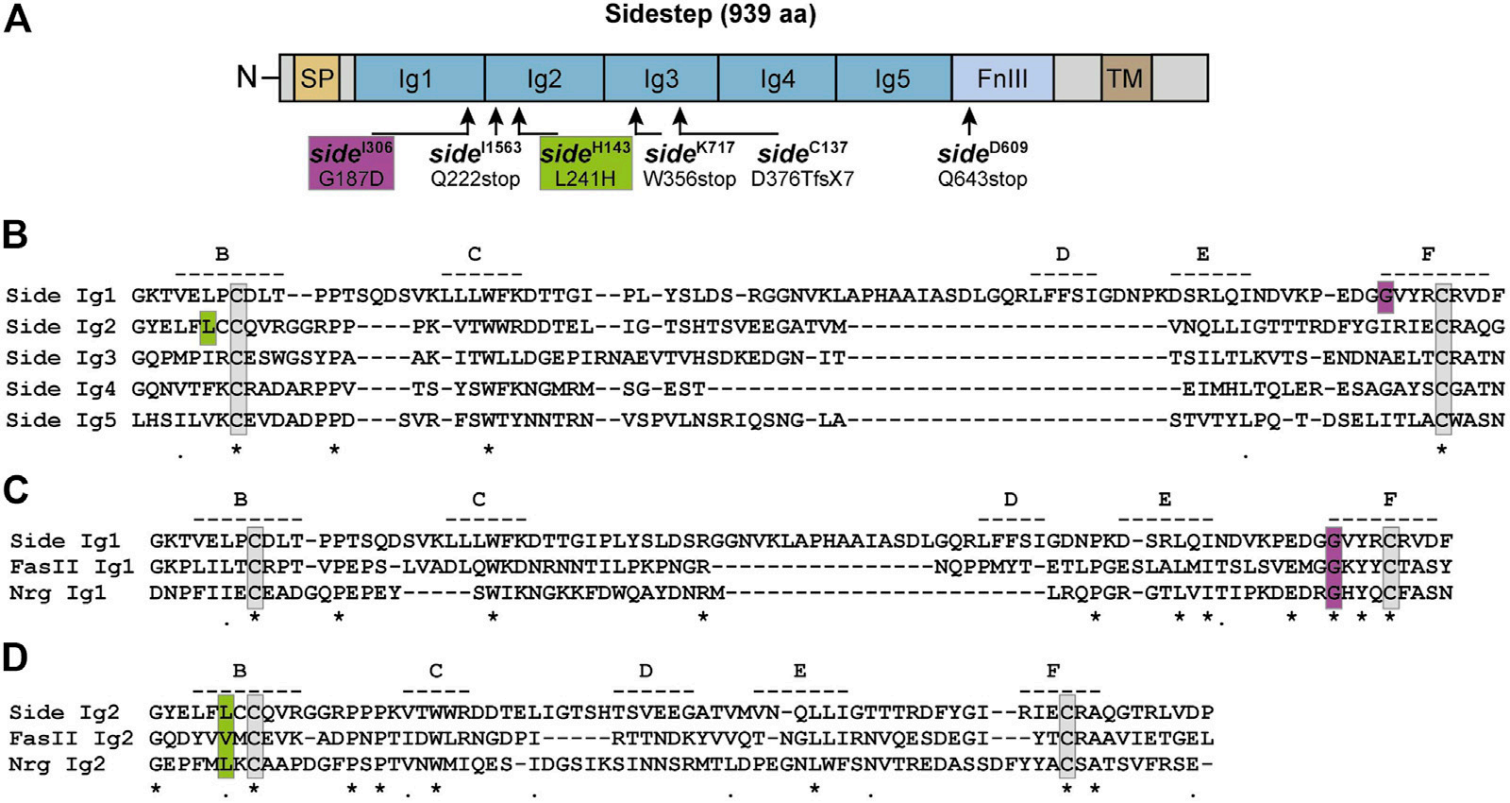
Single point mutations in *side* disrupt conserved amino acids. **(A)** Domain structure of Sidestep (Side). Arrows indicate positions of mutagen-induced point mutations. Alleles and their amino acid changes are indicated. aa, amino acids; fsX7, frame shift inducing stop codon after seven aa; SP, signal peptide; Ig, immunoglobulin domain; FnIII, fibronectin type-III domain; TM, transmembrane domain. Not to scale. **(B)** Sequence alignment of the five Ig domains of Side. The approximate positions of potential β-strands are indicated by dashed lines and labelled B through F. Strands A and G are not depicted. The D strand is misaligned due to a sequence insertion in Ig1. Identical and similar amino acids are marked by asterisks and periods, respectively. Essential cysteines are boxed (grey). The mutated glycine in *side* ^I306^ and leucine in *side* ^H143^ are boxed magenta and green, respectively. **(C)** Alignment of the first Ig domains of the structurally, but not functionally, related proteins Side, Fasciclin II (FasII) and Neuroglian (Nrg). Essential cysteines are boxed (grey). The mutated glycine in *side* ^I306^ is highly conserved (boxed, magenta).**(D)** Alignment of the second Ig domains of Side, FasII and Nrg. Hydrophobic amino acids are conserved at and next to the mutated leucine (boxed, green).

Several alleles of *sidestep* (*side* ^D282^, *side* ^D609^, *side* ^H143^, *side* ^P45^) were isolated in an embryonic mutagenesis screen of the III. chromosome based on their striking motor axon phenotypes (Sink et al., 2001). Four additional alleles (*side* ^C137^, *side* ^I306^, *side* ^I1563^, *side* ^K717^) were identified in a larval screen for mutants affecting the growth and morphology of neuromuscular junctions (NMJs) (Aberle et al., 2002; Siebert et al., 2009). All of these alleles carry EMS (ethyl methanesulfonate)-induced point mutations but have never been sequenced. To identify potentially informative amino acid changes, we sequenced available alleles on the genomic level, and *side* ^C137^ additionally on the transcript level (Figure 1). Sidestep is a 939 amino acid (aa) transmembrane protein of the immunoglobulin superfamily containing five extracellular immunoglobulin (Ig) domains and a fibronectin type-III (FnIII) domain (Figure 1A) (Sink et al., 2001; Li et al., 2017). Sequencing revealed three nonsense mutations truncating the protein prematurely (Q222stop in *side* ^I1563^, W356stop in *side* ^K717^, Q643stop in s*ide* ^D609^) (Figure 1A). The *side* ^C137^ allele had an alteration in the splice acceptor site of exon seven inducing a frame shift of -1 that resulted in D376T and a premature stop codon after seven additional amino acids (GISQRVS). Interestingly, we also found two missense mutations in conserved amino acids of Ig1 and Ig2 (G187D in *side* ^I306^, L241H in *side* ^H143^).

Ig-like repeats in Side and other family members are approximately 110 aa in length and composed of two β-sheets that typically consist of seven antiparallel β-strands, numbered A-G. The β-sheets are held together by two conserved cysteines that are spaced 40–60 aa apart and that form an intramolecular disulfide bond (boxed grey in Figure 1B). The G187D mutation (boxed magenta in Figure 1B) occurred in the first Ig domain at the -4 position with respect to the essential cysteine in the F-strand. Amino acids in this position greatly contribute to the stabilization of the fold (Bieber et al., 1989). Since Glycine 187 is highly conserved in related proteins such as Fasciclin II (FasII) or Neuroglian (Nrg) (Figure 1C), an exchange to aspartate likely affects folding of the first Ig domain. The L241H mutation affects the second Ig-domain and is located at the -2 position of the first cysteine in Ig2 (boxed green in Figure 1B). The leucine is oriented towards the hydrophobic core and substitution to a basic histidine likely destabilizes Ig2, too (Figure 1D). Thus, the identified point mutations are predicted to either truncate Side prior to the transmembrane domain or interfere with the architecture of its extracellular Ig domains.

### Mutations in *side* prevent axonal localization or truncate the protein prematurely

**FIGURE 2:**
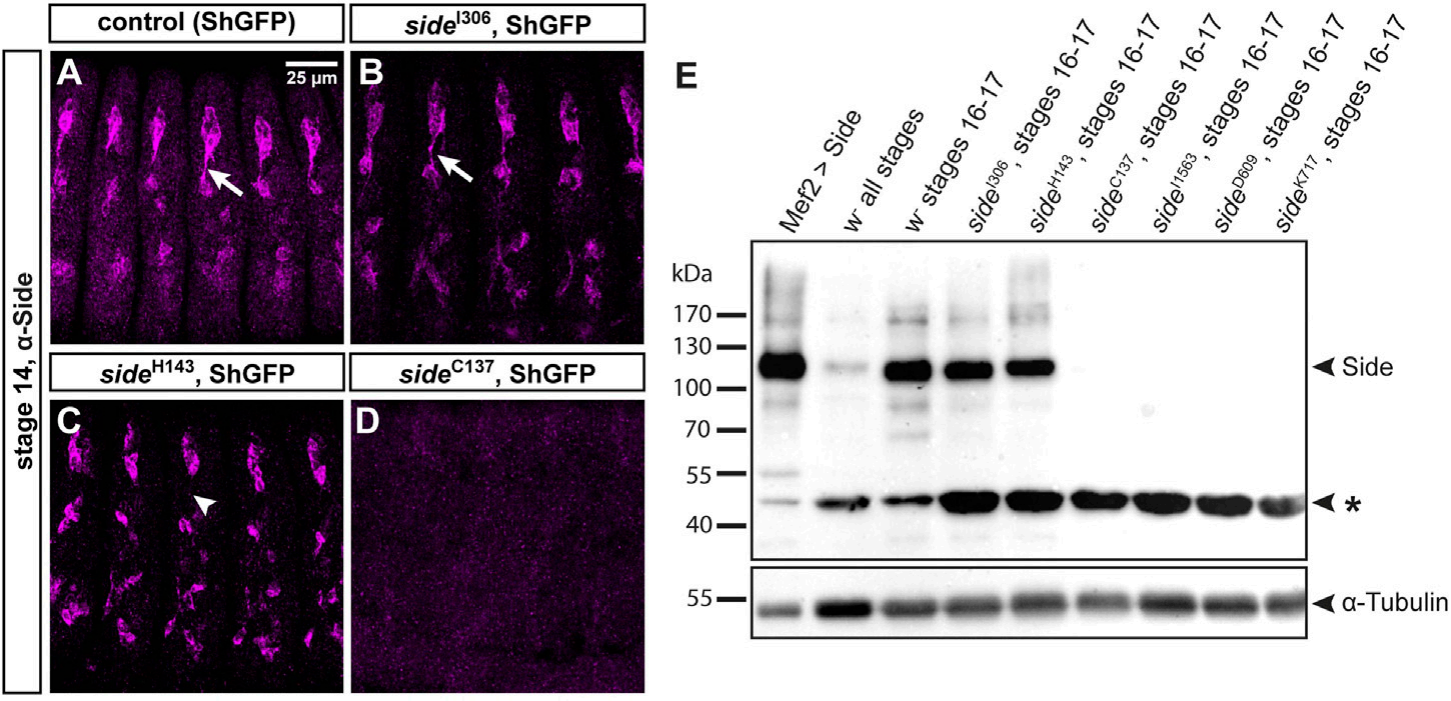
*Side* alleles carrying missense mutations express full-length Side.**(A-D)** Confocal images of ShGFP control and *side* homozygous mutant embryos (stage 14, lateral views) stained against Side (magenta). **(A)** In controls, Side is strongly expressed in developing sensory neurons and their outgrowing axons (arrow). **(B)** Expression level and subcellular location of Side are similar in *side* ^I306^ and controls. **(C)** In *side* ^H143^ mutants, Side is restricted to the soma and axonal localization is abolished (arrowhead). **(D)** Side is not detected in *side* ^C137^. Confocal images in this and the following figures are all maximum intensity projections. Anterior is left and dorsal is up. Scale bar: 25 μm. **(E)** Western blot of lysates from control and homozygous *side* mutant embryos developed with anti-Side antibodies. (Upper panel) Full-length Side migrates as a single band at 120 kDa. Lane 1: Exogenous, untagged Side is strongly expressed by Mef2-Gal4. Lanes 2–3: Embryo collections of all stages exhibit weaker Side signals than collections enriched for older embryos (st. 16–17, hand-sorted). Lanes 4–5: In *side* ^I306^ and *side* ^H143^, Side migrates at comparable size and expression level as endogenous Side. Lanes 6–9: Side is not detectable in *side* alleles carrying protein truncating mutations. Asterisk marks a suspicious band of unknown origin at approximately 47 kDa that cross-reacts with the monoclonal antibody and that seems to be enriched in all mutants. (Lower panel) The same blot developed with anti-α-Tubulin antibodies (loading control).

Since mutations identified in unbiased screens frequently affect functionally important domains, we wanted to analyze their effects on protein expression and/or subcellular localization. Side is recognized by a monoclonal antibody raised against the extracellular domain (aa 241–786, comprising Ig2-FnIII) (Sink et al., 2001). In control embryos, here isogenic ShGFP, also called ShakerGFP or CD8-GFP-Sh (Zito et al., 1999), Side was enriched in peripheral sensory neurons at stage 13–15 (Figure 2A). It was prominently expressed in the soma and developing axons but not detectable in dendrites. In homozygous *side* ^I306^ embryos, carrying the G187D exchange, Side localized to the plasma membrane and appeared indistinguishable from wild-type Side, suggesting that the mutation affects a function of Side that is independent of its subcellular localization or expression level (Figure 2B). Interestingly, in *side* ^H143^, having the L241H mutation, Side was strongly withheld in the soma and undetectable in axons (arrowhead in Figure 2C). This phenotype was also evident at later embryonic stages, suggesting that axonal localization was perturbed. Interestingly, mutant Side proteins were still expressed in sensory neurons at stage 17, when wild-type Side is normally no longer detectable in this tissue using anti-Side antibodies (Siebert et al., 2009). Constitutive expression of these mutant proteins might hint to defects in their posttranslational regulation. No Side protein was detectable in homozygous embryos carrying any of the premature stop mutations (Figure 2D).

We confirmed these results by Western blotting using lysates extracted from overnight egg collections or manually sorted *white* embryos (stage 16–17) (Figure 2E). The calculated molecular weight of Side (isoform A, 939 aa) was 101 kDa but the antibodies detected a prominent band at 115–120 kDa (Figure 2E). Compared to lysates of all stages, Side appeared to be enriched at older stages. Notably, mutant proteins in *side* ^I306^ and *side* ^H143^ migrated at equal heights and showed comparable expression levels as wild-type Side. Side was not detectable in any allele with premature stop codons (Figure 2E). The monoclonal antibody reacted also with a second, unidentified band at ∼45 kDa (asterisk in Figure 2E), which actually might be a specific reaction product, as its expression level was increased in all mutants, when compared to α-Tubulin loading controls. Attempts failed, however, to detect alternatively spliced transcripts encoding a protein of similar size. These results suggest that the first two Ig domains are relevant for Side function, as full-length proteins are expressed in alleles carrying missense mutations but are unable to guide motor axons.

### The first Ig domain of Side is functionally required to attract motor axons

**FIGURE 3:**
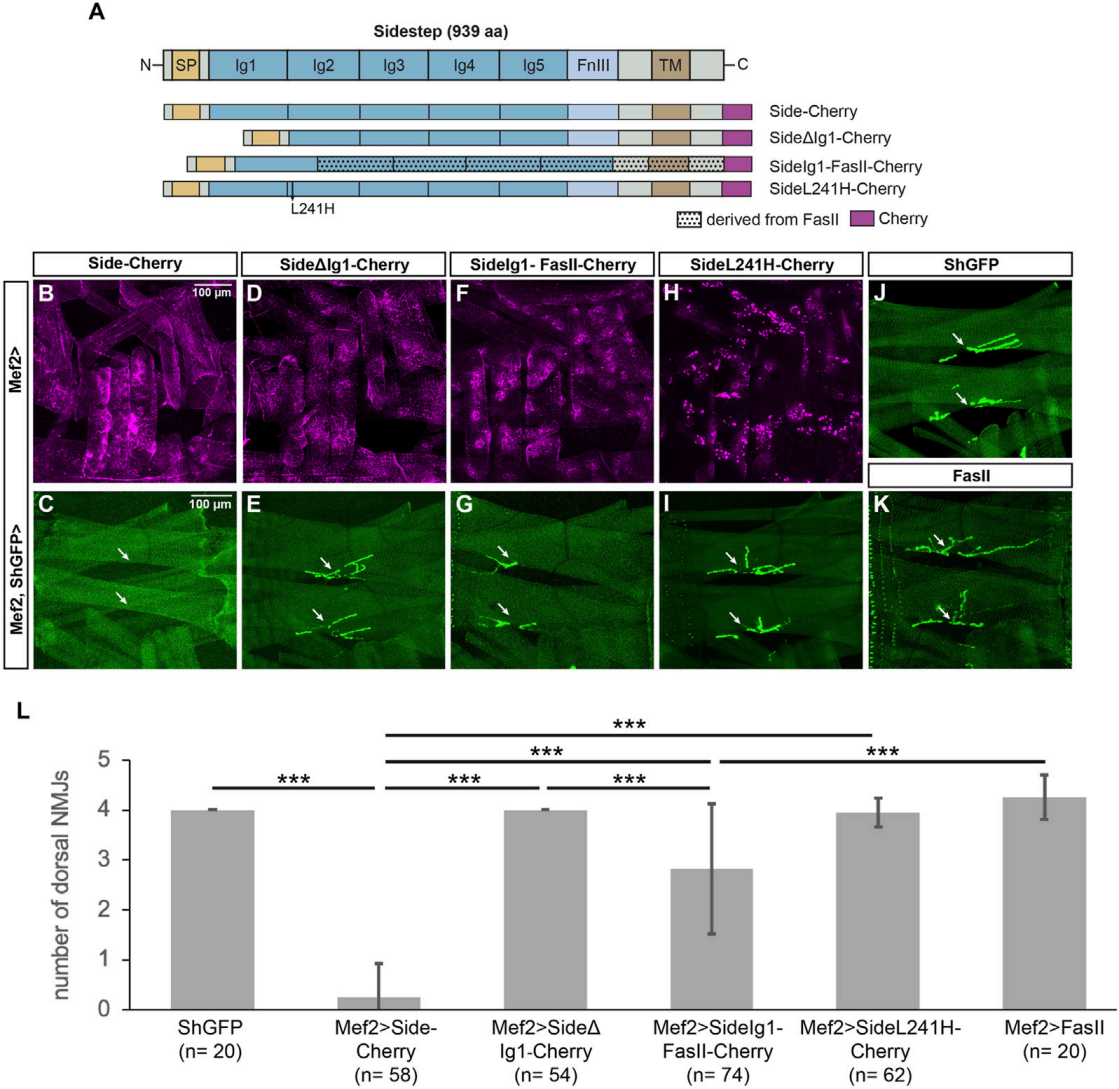
The first Ig domain of Side is necessary and partially sufficient to attract motor axons. **(A)** Scheme of the domain structure of Sidestep and various Cherry-fusion proteins used in the study. SideΔIg1 lacks the first Ig domain, and SideIg1-FasII replaces the first Ig domain in FasII with the first Ig domain of Side. The positions of the L241H point mutation and the Cherry tags (magenta) are indicated. aa, amino acids; SP, signal peptide; Ig, immunoglobulin domain; FnIII, fibronectin type-III domain; TM, transmembrane domain. FasII, Fasciclin II. Not to scale. **(B, D, F, H)** Confocal images of undissected third instar larvae expressing the indicated Cherry-fusion proteins in muscles under control of Mef2-Gal4. Depicted is the lateral muscle field showing the distribution of the fusion proteins in transverse muscles M21-24 (center, other muscles are also visible). **(C, E, G, I–K)** Confocal images of undissected ShGFP larvae showing that ectopic expression of Side-Cherry in muscles prevents NMJ formation on dorsal-most muscles 1/9 and 2/10 (C, arrows). Replacing the first Ig domain of FasII with the first Ig domain of Side disturbed dorsal NMJs in size and position (**G**, arrows). Compared to ShGFP controls **(J)**, expression of SideΔIg1 **(E)**, SideL241H **(I)** or FasII **(K)** had no effect. Scale bar: 100 µm. **(L)** Quantification of the number of NMJs on the dorsal-most muscle pairs 1/9 and 2/10 in the indicated genotypes. ShGFP control animals have four NMJs in this region, one major structure per muscle fiber. n, number of hemisegments; statistical significance was calculated using two-tailed Mann–Whitney-U-test: ***, *p* < 0.001.

To directly test the function of the distal-most Ig domains in Side, we constructed Cherry-tagged deletion constructs (Figure 3A). High expression of exogenous Side or Side-Cherry in developing muscles during embryogenesis prematurely attracts outgrowing motor axons to undifferentiated myotubes, thus inhibiting their dorsal migration (Kinold et al., 2018). Dorsal-most muscles are hence not reached by the intersegmental nerve (ISN) and permanently lack NMJs (Kinold et al., 2018). As expected, when we expressed full-length Side-Cherry under control of Mef2-Gal4, dorsal muscles 1/9 and 2/10 almost completely lacked NMJs (0.26 ± 0.66 NMJs, n = 58) (Figures 3B,C, quantified in L). In contrast, wild-type or ShGFP control larvae have 4.00 ± 0.00 NMJs (*n* = 20) in this region (Figure 3J). Overexpression of SideΔΙg1-Cherry did not prevent NMJ formation, indicating that it is unable to attract motor axons (4.00 ± 0.00 NMJs, *n* = 54) (Figures 3D,E). Since both proteins reached the muscle surface, at least partially, and were expressed at similar levels (Figures 3B,D), the first Ig domain seemed to mediate this attraction. To test if it is also sufficient, we replaced the first Ig domain of Fasciclin II (FasII), a homophilic adhesion protein containing five extracellular Ig domains, with the first Ig domain of Side. Although FasII is expressed on motor axons, overexpression of full-length FasII in muscles did not prevent NMJ formation on dorsal muscles in this attraction assay (Figure 3K, number of NMJs 4.25 ± 0.44, n = 20) (Kinold et al., 2018). However, the chimeric fusion protein SideIg1-FasII-Cherry interfered with NMJ formation (2.82 ± 1.31 NMJs, *n* = 74) (Figures 3F,G), albeit less strongly than full-length Side (Fig. 3L). In contrast, Side carrying the L241H point mutation in Ig2 formed intracellular aggregates (see below) and had no effect on NMJ formation (3.95 ± 0.28 NMJs, n = 62) (Figures 3H,I). Taken together, these results indicate that the first Ig-domain of Side is necessary and partially sufficient to attract motor axons.

### Exogenous Beat functionally interacts with Side in muscles

**FIGURE 4:**
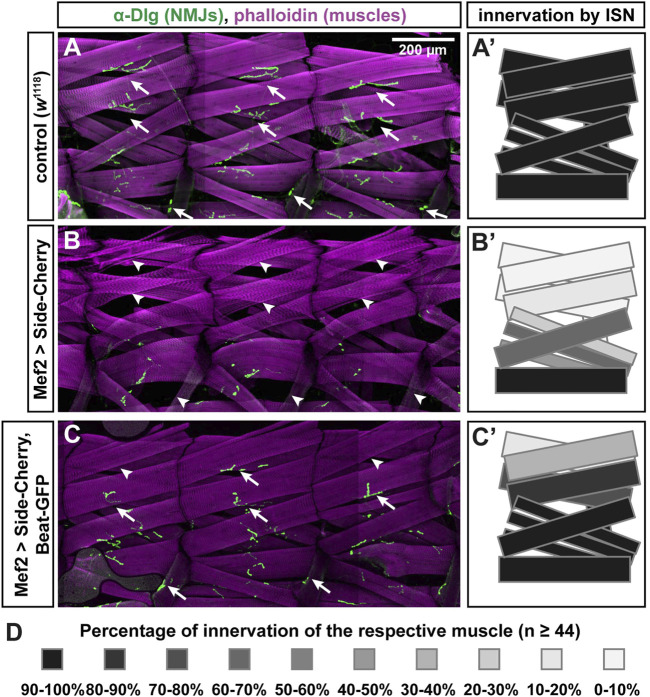
Beat-GFP suppresses innervation defects caused by overexpression of Side-Cherry. **(A-C)** Confocal images (tile scans) showing three abdominal hemisegments of larval fillet preparations stained with phalloidin (magenta) and anti-Discs large (Dlg) antibodies (green, originally recorded at 647 nm). Arrows indicate neuromuscular junctions (NMJs) at muscles 1/9, 2/10 and 8. Arrowheads mark the respective non-innervated fibers. (A) In *w* ^1118^ control larvae, each muscle fiber (magenta) is innervated by at least one NMJ (green). **(B)** Overexpression of Side-Cherry in all muscles prevented formation of NMJs on dorsal muscles and, to a lesser extent, also on lateral muscles. **(C)** Dorsal and lateral NMJs re-form when Side function is blocked by simultaneous co-expression of Beat-GFP (arrows). Arrowheads indicate fibers that still lack NMJs. Scale bar: 200 μm. **(A'–C')** Quantitative analysis of muscles innervated by the ISN. Muscles 1, 2, 3, 4, 9, 10, 11, 18, 19 and 20 were evaluated for the presence or absence of Dlg-positive NMJs. Innervation percentages for a given muscle fiber were coded in grey shades. Black indicates 90–100% innervation of a given muscle fiber by its NMJ, the lightest grey indicates 0–10% innervation. Re-appearance of dark colors in **(C')** shows the improvement of innervation. **(D)** Color-code indicating percentage of innervation of a given muscle. Black 100%, every muscle fiber evaluated is innervated; White 0%, none of the muscles is innervated; *n* = 44 or more hemisegments for each muscle.

Since Side interacts with Beaten path (Beat) (Siebert et al., 2009), it is possible that binding to Beat is lost in these missense alleles. To develop a functional assay for Beat-Side interactions, we took advantage of the extremely strong phenotype caused by Side-Cherry and stained muscles and NJMs in third instar larvae using phalloidin, an F-actin binding probe, and the postsynaptic marker Discs large (Dlg), respectively. Compared to *w* ^1118^ controls (Figures 4A–A′), overexpression of Side-Cherry using Mef2-Gal4 caused complete absence of Dlg-positive NMJs (green) on most, if not all, dorsal muscle pairs 1/9 and 2/10 (Figure 4B-B′). In contrast, simultaneous co-expression of Beat-GFP and Side-Cherry, however, suppressed this gain-of-function phenotype, resulting in a much higher percentage of NMJs in dorsal body wall regions (Figure 4C-C′). With the exception of dorsal-most muscles 1 and 9, quantification showed that muscles in the ISN target field were innervated close to wild-type levels (Figure 4D). Thus, Side-Cherry and Beat-GFP functionally interact in muscles, which could be exploited to determine the binding sites.

### The first immunoglobulin domain of Beat mediates the interaction with Side

**FIGURE 5:**
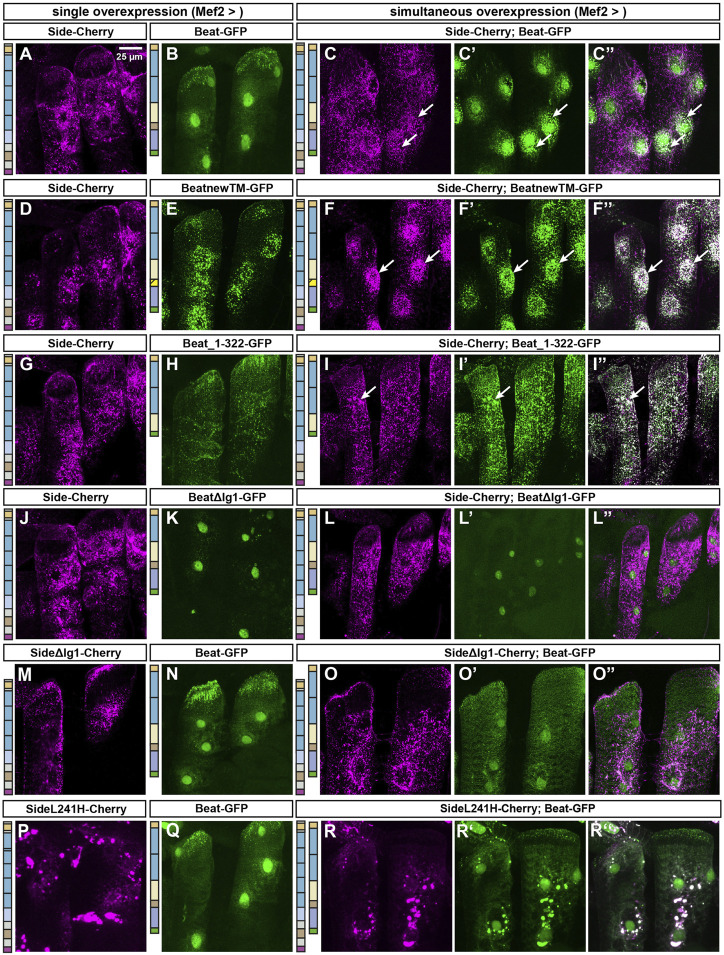
The first immunoglobulin domains of Beat and Side mediate their mutual interaction. **(A–Q)** Confocal images showing lateral muscles 21 and 22 in third instar larvae imaged through the translucent cuticle of intact third instar larvae using a ×63 oil objective. The two columns to the left show the distribution of either Side-Cherry (magenta) or a given Beat fusion protein (GFP, green) expressed individually in muscles. The three columns to the right show simultaneous co-overexpression of the indicated combination of two constructs. **(A–C'')** Side-Cherry forms punctate aggregates but reaches also the muscle surface **(A)**. Beat-GFP mostly accumulates in muscle nuclei **(B)**. Simultaneous co-overexpression of both proteins re-distributes Beat-GFP to cytoplasmic aggregates surrounding nuclei, where portions of both proteins co-localize (arrows in C-C''). **(D–F'')** BeatnewTM-GFP completely traps Side-Cherry in intracellular particles surrounding nuclei (arrows). **(G–I'')** Beat_1–322-GFP co-localizes with Side-Cherry in punctate clusters throughout the sarcoplasm (arrows). **(J–L'')** BeatΔIg1-GFP accumulates in muscle nuclei, irrespective of whether or not Side-Cherry is co-expressed. **(M–O'')** SideΔIg1-Cherry shows a similar subcellular distribution as full-length Side **(M)**. Co-expression with Beat-GFP results only in a few Beat-GFP punctae outside nuclei **(O–O'')**. **(P–R'')** Side carrying the L241H point mutation clusters in large sarcoplasmic aggregates that do not reach the muscle surface **(P)**. In co-overexpression experiments, Beat-GFP accumulates in these aggregates but enriches also in nuclei. Schemes to the left of each column indicate the domain structure of the constructs used for each experiment.Scale bar: 25 μm.

To directly visualize Beat-Side interactions *in vivo*, we first created a series of Beat C-terminal deletion constructs (see Figure 7A) but then targeted also potentially functional domains such as the transmembrane domain or the immunoglobulin domains (see Figure 8A). We tagged these constructs with GFP at their C-termini and expressed them either individually or simultaneously with Side-Cherry in larval muscles. While Side-Cherry localized predominantly to numerous aggregates in the cytoplasm but also at the plasma membrane (Figure 5A), Beat-GFP accumulated in nuclei (Figure 5B). Co-expression of both proteins partially trapped Beat-GFP in ER cisternae, visible as punctate aggregates surrounding nuclei (arrows in Figure 5C-C‴). BeatnewTM-GFP interacted strongly with Side-Cherry, withholding it completely in the ER, which resulted in precise co-localization at this level of magnification (Figure 5D–F″). Co-localization of Beat_1–322-GFP and Side-Cherry was also evident but occurred in the entire cytoplasm and at the muscle surface but not around nuclei, indicating that the C-terminus of Beat was not required for the interaction, and that these complexes bypass trafficking controls in the ER (Figures 5G–I″). BeatΔIg1-GFP was weakly expressed in muscles. Upon co-expression with Side it remained in the nucleus and did not show any sign of protein-protein interaction (Figure 5J–L″). Conversely, Side lacking its first Ig domain did not form punctate aggregates with Beat-GFP around muscle nuclei, indicating lack of interactions (Figures 5M–O″). SideL241H strongly aggregated in intracellular compartments, which were also enriched for Beat-GFP, indicating that delivery to the plasma membrane is inhibited but interaction with Beat is still possible (Figures 5P–R″).

To confirm some of these interactions *in vitro*, we produced protein lysates from larval fillets for co-immunoprecipitation experiments. Precipitation of Beat-GFP using anti-GFP antibodies co-purified Side-Cherry from body wall lysates of larvae overexpressing these proteins in muscles (Supplementary Figure S1). Similarly, BeatnewTM and Beat_1–322 but not BeatΔIg1 co-precipitated Side-Cherry (Supplementary Figure S1), further strengthening the idea that the first Ig domain of Beat mediates the interaction.

**FIGURE 6:**
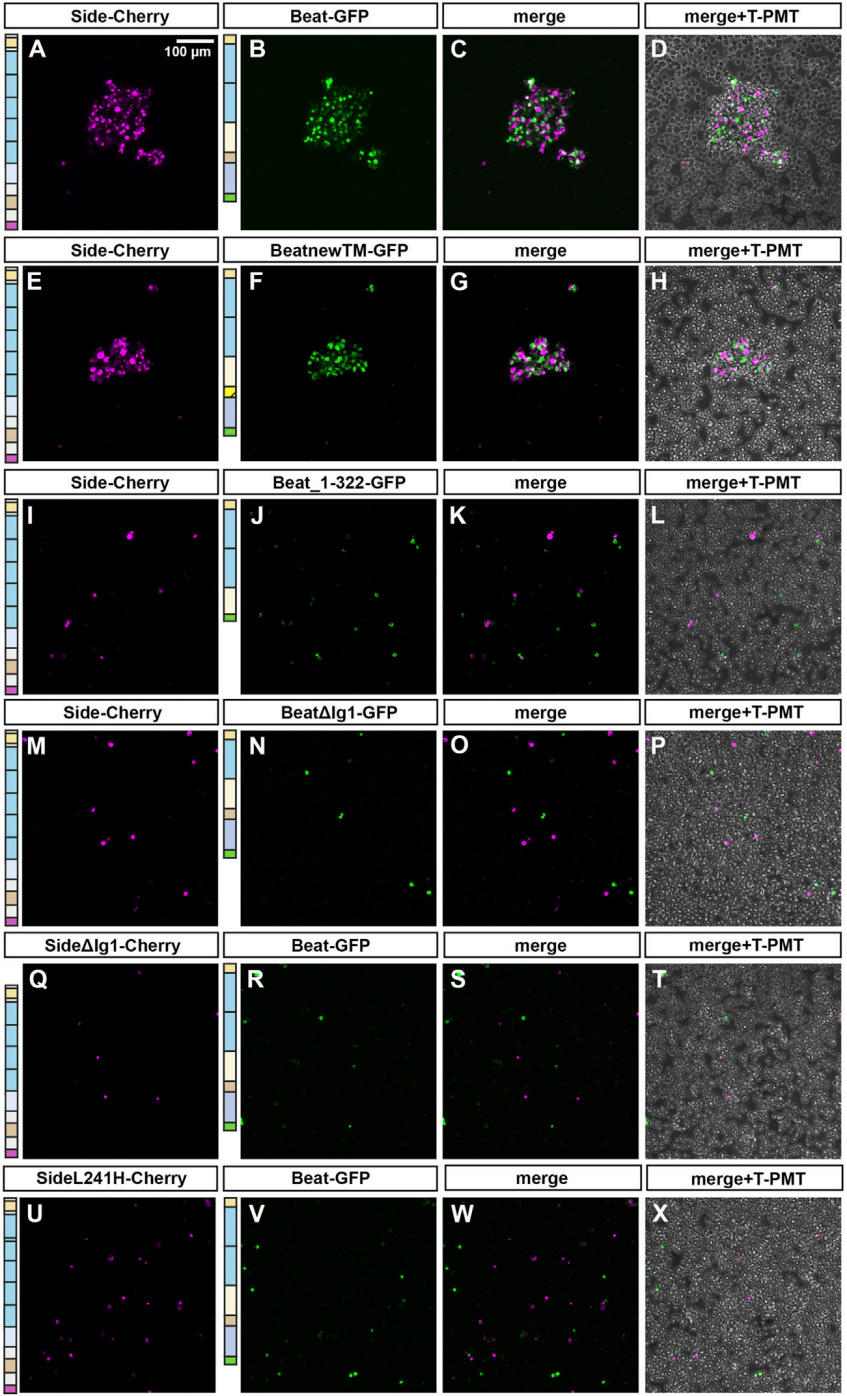
The first Ig domain of Beat and Side is required to mediate cell-cell interaction in S2 cells. **(A–X)** S2 cells were transiently transfected with one of the indicated constructs in separate dishes. After 3 days, cell suspensions were mixed and subjected to cell aggregation assays followed by confocal microscopy. Images in the column to the very right show in addition untransfected cells. **(A–D)** S2 cells expressing Side-Cherry and Beat-GFP sort themselves out from untransfected cells and form large cell aggregates. **(E–H)** BeatnewTM-GFP expressing cells interact with Side-Cherry expressing cells. **(I–L)** Beat_1–322 fails to form cell-cell aggregates with Side-Cherry transfected cells, likely because it is either secreted or not firmly attached to the cell surface. **(M–P)** BeatΔIg1-GFP does not interact with Side-Cherry-expressing cells. **(Q–T)** Similarly, SideΔIg1-Cherry is not able to form aggregates with Beat-GFP. **(U–X)** SideL241H-Cherry also fails to interact with Beat-GFP-expressing cells. Scale bar: 100 μm.

We also verified these interactions in an independent cell culture system using transiently transfected S2 cells. Normally a suspension of non-adherent cells, S2 cells transfected with full-length Beat formed large cell clusters, only when mixed together with Side-Cherry expressing S2 cells (Figures 6A–D). Note that transfected cells sort out themselves from untransfected cells. Similarly, BeatnewTM-expressing cells tightly bound to Side-Cherry-expressing cells (Figures 6E–H). However, Beat_1–322 failed to induce clustering, probably because it is secreted and not anchored on the cell surface (Figure 6I-L). Quantitative measurements showed that the average surface area of these clusters was clearly reduced compared to Beat- or BeatnewTM-induced clusters (Supplementary Figure S2). Cell-cell aggregates were similarly completely abolished, when the first Ig domain was deleted in Beat (Figures 6M–P) or Side (Figures 6Q–T). Similarly, the L241H mutant protein, having a point mutation in the second Ig domain, failed to interact with Beat-transfected cells, likely due to a failure to reach the cell surface (Figures 6U–X). Thus, these results suggest that Beat and Side interact *via* their first Ig domains, and deleterious mutations in this domain abrogate complex formation and attraction of motor axons.

### C-terminal domains of Beat accumulate in muscle nuclei

**FIGURE 7:**
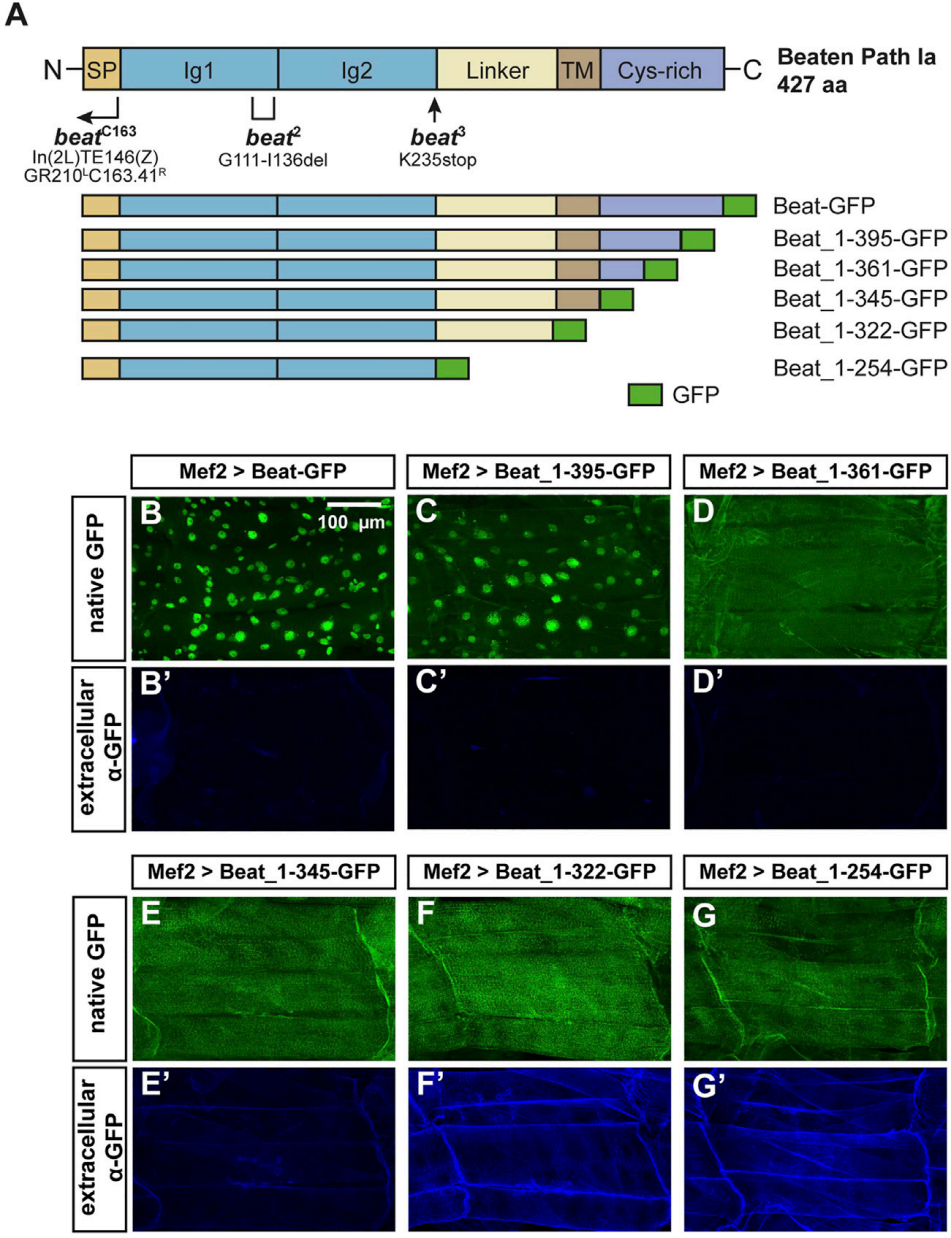
N-terminal domains of Beat are exposed on muscle surfaces.**(A)** Upper panel: Domain structure of Beat (427 aa). Bracket shows the extent of the deletion in *beat* ^2^. Arrow indicates position of the point mutation in *beat* ^3^. Right-angled arrow marks the approximate breakpoint of the inversion in *beat* ^C163^ (Fambrough and Goodman, 1996). SP, signal peptide; Ig, immunoglobulin domain; Linker, sequence enriched in glycine and serine residues. TM, potential transmembrane domain; Cys-rich, cysteine-rich domain. Not to scale. Lower panel: Schemes of Beat C-terminal deletions used in this study. Amino acids of Beat are indicated by numbers, GFP was inserted at the C-terminus. **(B–G')** Confocal images of non-permeabilized fillet preparations of third instar larvae expressing the indicated Beat constructs in muscles under control of Mef2-Gal4. **(B–G)** Native GFP fluorescence of the fusion proteins. **(B'–G')** Extracellular GFP as detected by anti-GFP antibodies. **(B–C')** GFP tags of Beat-GFP and Beat_1–395-GFP accumulate in nuclei but not on muscle surfaces. **(D–E')** Beat_1–361-GFP and Beat_1–345-GFP distribute homogenously but GFP remains intracellularly. **(F–G')** Beat_1–322-GFP and Beat_1–254-GFP lacking the potential transmembrane domain and the Cys-rich region expose GFP extracellularly.

To search for further functional domains in Beat, we first sequenced available *beat* alleles for potential informative, EMS-induced point mutations. Full-length Beat is a 427 amino acid (aa) protein consisting of two N-terminal Ig domains and a cysteine-rich C-terminus with similarities to cysteine-knots (Figure 7A) (Fambrough and Goodman, 1996; Bazan and Goodman, 1997; Mushegian, 1997). In addition, domain structure predictions delineate a flexible linker region and a potential transmembrane domain (Figure 7A) (Siebert et al., 2009). In *beat* ^2^ (van Vactor et al., 1993), we discovered a 26 aa in-frame deletion (G111-I136) and in *beat* ^3^ a nonsense mutation in lysine 235 (Figure 7A). Since *beat* ^C163^ is an inversion with a breakpoint in the 5′ region of the gene (Fambrough and Goodman, 1996), all of these mutations are therefore predicted to abolish *beat* function.

Next, we constructed a series of C-terminal deletion constructs tagged with GFP (Figure 7A) and expressed them in muscles using Mef2-Gal4. As Beat is normally expressed in motoneurons, muscle expression might affect their function but due to a large size, muscles provide an excellent subcellular resolution and experimental accessibility. To our surprise, Beat-GFP and Beat_1–395-GFP accumulated in muscle nuclei (Figures 7B,C). The remaining constructs were homogenously distributed in muscle fibers (Figures 7D–G), indicating that a C-terminal motif might trigger nuclear accumulation.

To test the orientation of Beat relative to the muscle membrane we took advantage of GFP as a specific antibody epitope. If Beat is a standard type-I transmembrane protein, its C-terminus should not be accessible for antibodies in larval fillets under non-permeabilizing conditions. In fact, anti-GFP antibodies failed to detect Beat-GFP on the muscle surface in the absence of detergent (Figure 7B′). Since GFP in nuclei remained unstained, this method seemed to be suitable for distinguishing extracellular from intracellular GFP. Shorter constructs also did not expose GFP on the muscle surface (Figure 7C′–E′). However, C-termini of Beat_1–322-GFP and Beat_1–254-GFP were clearly detected on the extracellular side of the muscle membrane and co-distributed with their native fluorescence (Figure 7F′–G′). Thus, only Beat proteins lacking the potential transmembrane domain expose C-terminal GFP on muscle surfaces.

### Prominent role for the potential transmembrane domain in sorting Beat to different subcellular compartments

**FIGURE 8:**
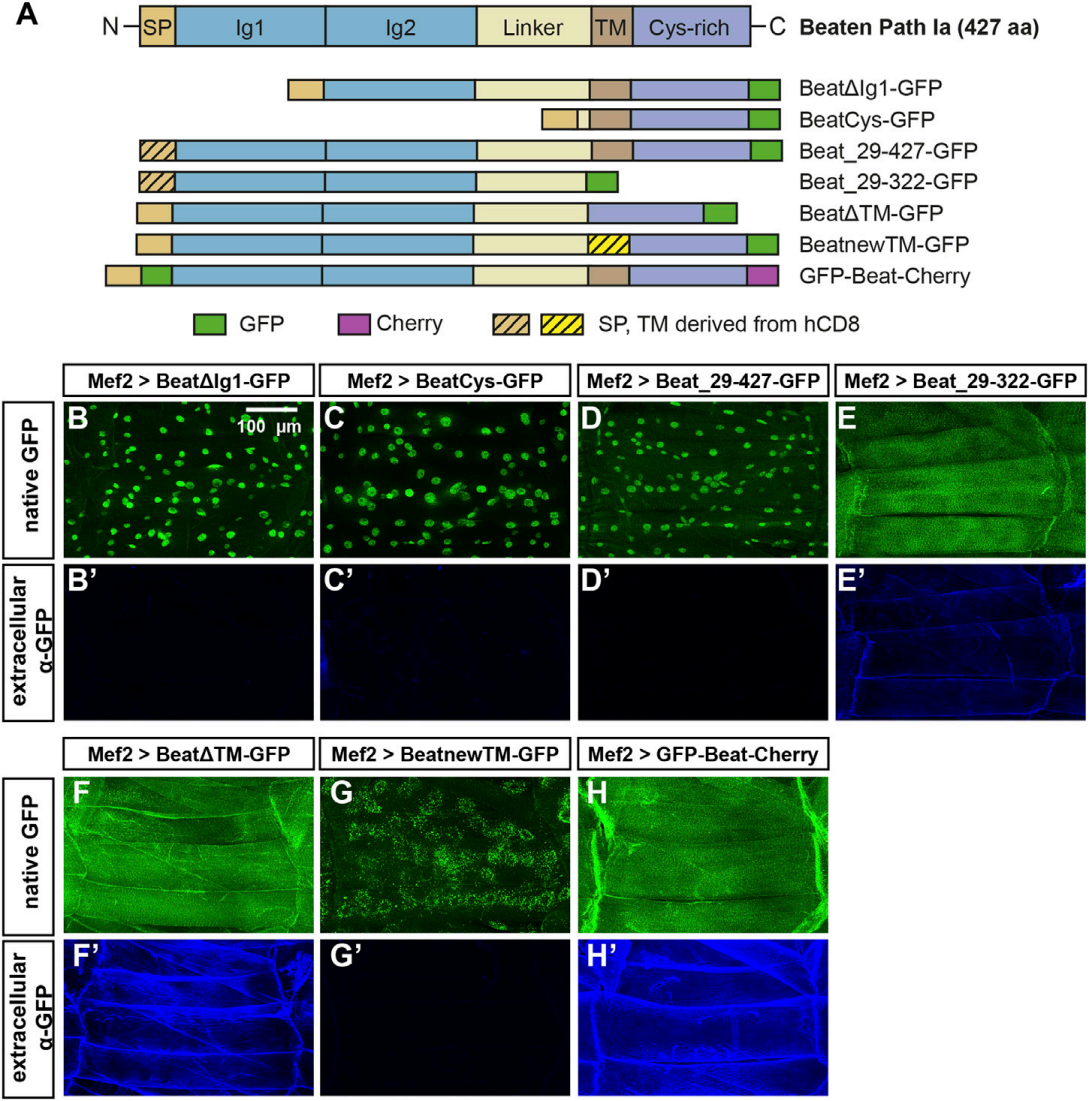
The potential transmembrane domain and the Cys-rich domain are sufficient for nuclear import. **(A)** Domain structure of wild-type Beat (427 aa) and Beat constructs used in this study. Encoded amino acids of Beat and the positions of fluorescent tags (GFP, green; Cherry, magenta) are indicated. Sequences derived from human CD8 are hatched. Other abbreviations as in Figure 7. Not to scale. **(B–H')** Confocal images of non-permeabilized fillet preparations of third instar larvae expressing the indicated Beat constructs in muscles under control of Mef2-Gal4. **(B–H)** Native GFP fluorescence. **(B'–H')** Extracellular GFP as detected by anti-GFP antibodies. **(B–C')** Deletion of the first or the first two Ig domains causes C-terminal GFP to accumulate in muscle nuclei but not on muscle surfaces. **(D–E')** Replacement of the endogenous signal peptide by a generic version from human CD8 does neither affect nuclear localization (Beat_29–427-GFP) nor extracellular exposure of GFP (Beat_29–322-GFP). **(F–G')** Deletion of the transmembrane region in BeatΔTM-GFP leads to exposure of GFP on muscle surfaces **(F–F)**. Exchange of the transmembrane domain traps BeatnewTM-GFP in intracellular, perinuclear ER compartments **(G)**. **(H–H')** GFP fused to the N-terminus of Beat distributes homogenously on muscle surfaces.Scale bar: 100 μm.

To test if nuclear accumulation is dependent on the extracellular Ig domains, we constructed two N-terminal deletions (Figure 8A). Expression of both BeatΔIg1-GFP and BeatCys-GFP in muscles resulted in nuclear localization of GFP without any surface exposure (Figure 8B-C′). We also investigated if the predicted signal peptide has a potential function in sorting or anchoring Beat to the cell surface and replaced it with one originating from human CD8 (Figure 8A). Expression of Beat_29–427 in muscles, however, showed that this exchange did not influence nuclear import (Figure 8D-D′). Similarly, significant amounts of Beat_29–322-GFP were still attached to the muscle surface (Figure 8E-E′, compare to Figure 7F-F′). Since the N-and C-terminal deletion constructs point to a role of the potential transmembrane domain in the subcellular distribution of Beat, we deleted it in BeatΔTM-GFP. Although the entire C-terminus was present, GFP did not enrich in nuclei but instead was exposed on the muscle surface (Figure 8F-F′). Insertion of an ectopic transmembrane derived from human CD8 trapped BeatnewTM-GFP in intracellular compartments that prominently surrounded all muscle nuclei (Figure 8G-G′). Probing these punctate aggregates with an ER marker showed co-localization to a large extent (Supplementary Figure S3). The combined results of these experiments indicate that the N-terminus of Beat extends into the extracellular space. We therefore constructed a Beat construct that is labelled on both its N- and C-terminus with GFP and Cherry, respectively, giving rise to GFP-Beat-Cherry. Following its expression in muscles, GFP was now indeed homogenously distributed on the extracellular surface and no longer enriched in nuclei (Figure 8H-H′). This was confirmed by independent experiments using the deGradFP system (Caussinus et al., 2011). When GFP resided in extracellular compartments, like in GFP-Beat-Cherry, the fusion proteins were protected from degradation and neither their expression level nor their subcellular distribution changed (Supplementary Figure S4). In contrast, fusion proteins like BeatnewTM-GFP exposing GFP in the cytoplasm were strongly downregulated. Thus, N- and C-termini of Beat might separate during posttranslational processing steps.

### The N- and C-termini of Beat separate in neurons, muscles and cultured cells

**FIGURE 9:**
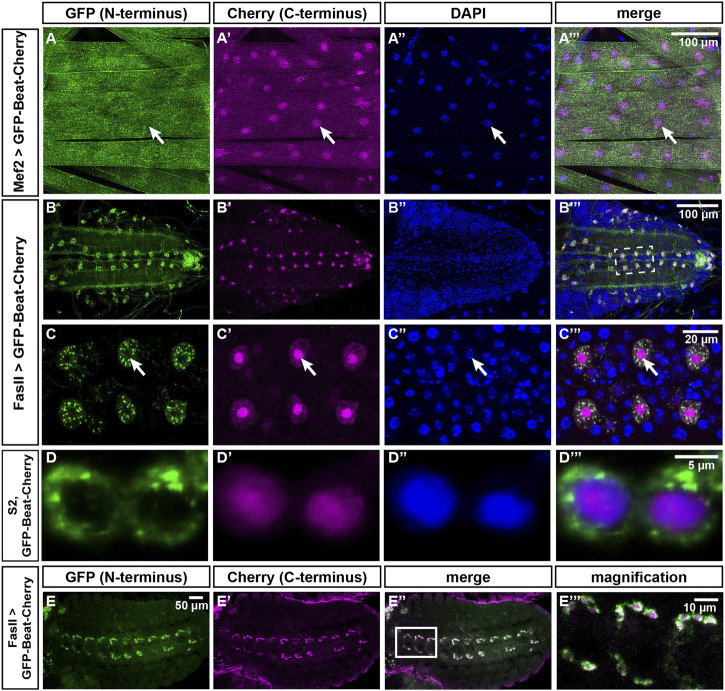
The N- and C-termini of Beat localize to different subcellular compartments. **(A–C''')** Confocal images of dissected third instar larvae expressing GFP-Beat-Cherry in muscles or motor neurons. Native GFP (green), native Cherry (magenta), DAPI (blue). **(A–A''')** Under control of Mef2-Gal4, N-terminal GFP distributes over the entire muscle surface, while C-terminal Cherry is predominantly nuclear (arrows). **(B–B''')** Ventral nerve cord (dorsal view) showing expression of GFP-Beat-Cherry in motor neurons using FasII-Gal4. **(C–C''')** White frame in **(B''')** enlarged. Arrows label a single RP motor neuron, showing punctate GFP in the cytoplasm that does not overlap with nuclear Cherry. **(D–D''')** Confocal images of transiently transfected S2 cells expressing GFP-Beat-Cherry. While N-terminal GFP accumulates predominantly in the cytoplasm **(D)**, C-terminal Cherry **(D')** co-localizes with nuclear DAPI **(D'')**. Merged images **(D''')**. **(E-E''')** Expression of GFP-Beat-Cherry under control of FasII-Gal4 in stage 15 embryos (ventral view). White frame in **(E'')** is enlarged in **(E''')**. GFP and Cherry separate already when motor axons actively migrate into the periphery.Scale bars: 5, 10, 20 and 100 μm as indicated.

To directly visualize the supposed separation of the N- and C-terminus of Beat, we analyzed the distribution of GFP-Beat-Cherry in larval muscles (Figures 9A–A‴). While GFP was homogenously distributed on the muscle surface, Cherry was concentrated in nuclei. To test if this separation occurs also in motor neurons, we expressed GFP-Beat-Cherry under control of FasII-Gal4. N- and C-termini were already separated in embryos at stage 15, visible as a peripheral GFP-expressing domain separated from a central Cherry-expressing zone (Figure 9E-E‴). In third instar larvae, FasII-Gal4 was still expressed in a single RP motor neuron per hemineuromere (Figure 9B). While GFP was now distributed all over the soma and axons, Cherry was restricted to much smaller areas. Co-staining with DAPI demonstrated that these are nuclei (Figure 9B′-B‴). Higher magnification clarified that punctate cytoplasmic GFP did not overlap with nuclear Cherry (Figure 9C-C‴). We also tested GFP-Beat-Cherry in transiently transfected S2 cells and found that N- and C-termini accumulated in either cytoplasmic or nuclear compartments, respectively (Figure 9D-D‴). Thus, separation of the N- and C-termini of Beat occurs in several cell types, including motor neurons.

We tested all our Beat constructs for their ability to rescue transheterozygous *beat* ^3^/*beat* ^C163^ mutants. While any full-length construct did rescue the axon guidance and innvervation phenotypes up to 100%, all deletion constructs as well as the absence (BeatΔTM) or exchange (BeatnewTM) of the potential transmembrane domain completely failed to rescue *beat* mutants. Rescue was only possible when neuronal drivers Elav-Gal4 or FasII-Gal4 were used (Supplementary Figure S5, quantified in S6). In light of a potential separation of Beat N- and C-termini, it is interesting to note that simultaneous expression of a combination of BeatCys-GFP lacking the N-terminus and Beat_1–345-GFP lacking the C-terminus also failed to rescue (Supplementary Figures S5, S6). However, due to the large size of these tags, we cannot fully exclude the possibility that they might hinder a function of Beat in one of the deletion mutants.

## Discussion

Motor axon guidance phenotypes in *beat* and *side* cause among the strongest and most penetrant neuromuscular wiring defects known in *Drosophila*. To better understand the molecular function of these guidance molecules, we performed a structure-function analysis. First, we sequenced available *beat* and *side* alleles and second, we constructed a series of deletion constructs which we tested for alterations in their subcellular localization and binding properties. The results show that the first immunoglobulin domain mediates the heterophilic interaction between Beat and Side. In particular, the exchange of a glycine residue in the first Ig domain of Side (G187D in *side* ^I306^) is informative, as it is conserved in Side paralogs and in distantly related adhesion proteins, like Fasciclin II and Neuroglian. SideG187D was expressed on the cell surface but lacked Side function. Based on its position near an invariant cysteine residue, the mutation likely disrupts the stability of three-dimensional structure of this domain.

We also identified a small in-frame deletion in *beat* ^2^ that is predicted to disrupt the first Ig domain and thus likely abolishes interaction with Side. However, it might also affect posttranslational processing or trafficking to growth cones, as abnormal Beat accumulations have been noticed in the soma of motor neurons (Fambrough and Goodman, 1996). Abnormal subcellular distribution of Side was also prominent in *side* ^H143^, having a point mutation in the second Ig domain that inhibited Side delivery into axons of sensory neurons and the plasma membrane in muscles. Since motor axons migrate along Side-expressing sensory axons (Siebert et al., 2009), this scenario could delay or confuse motor axons. Interestingly, both *side* ^H143^ and *side* ^I306^ are still expressed in sensory neurons at the end of embryogenesis (stage 17), when wild-type Side is no longer detectable by Side-specific antibodies. This downregulation depends on Beat (Siebert et al., 2009). Thus, these point mutations also interfere with controlled posttranslational regulation of Side.

None of the Side proteins carrying a premature stop codon was detected in Western blots or immunohistochemistry. Surprisingly, however, these Western blots revealed that endogenous Side was expressed at its highest levels at the end of embryogenesis at stage 16–17. Previous immunohistochemical stainings showed that Side is downregulated in peripheral nerves in late stage embryos, because it was no longer detected there by the very same antibody (Siebert et al., 2009). One possibility that might resolve this apparent contradiction, is the enrichment of Side in the neuropil at late stages (Sink et al., 2001). Alternatively, the epitope might be masked in peripheral nerves by formation of stable protein complexes, possibly with its receptor Beat, that are resolved in SDS-containing polyacrylamide gels but not in fixed tissues.

Endogenous Beat is specifically expressed in motor neurons (Fambrough and Goodman, 1996). Several of our Beat constructs, however, could not be detected in embryonic axons. To gain insights into their function and subcellular distributions, we expressed them in developing muscles. We are aware that one or several of the constructs might behave differently in neurons, but the full-length proteins functionally interacted in muscles, as Beat-GFP suppressed the attractiveness of Side-Cherry. This suppression is probably not due to titration of Gal4 as we have not seen a drastic reduction in Side levels upon co-expression of Beat-GFP (compare Figure 5A with 5C) but rather due to an interaction of these proteins in “cis”. However, interactions in “trans” might also be possible at the level of ER cisternae, diffusing vesicles and/or other dynamic membrane compartments if they come into close proximity of each other. Regardless of the precise interaction mechanism, we observed retention of Beat-Side complexes in the ER by wild-type and BeatnewTM-GFP, which should reduce the number of Side molecules at the plasma membrane followed by a reduction in axonal attraction.

Capture of Side-Cherry by BeatnewTM-GFP in the ER suggests that the potential transmembrane domain of endogenous Beat is important for sorting and/or processing of Beat. Replacement of the potential transmembrane region of endogenous Beat by one derived from human CD8, trapped BeatnewTM in the ER, and so was Side-Cherry. In contrast, Beat_1–322-GFP lacking this domain and the Cys-rich C-terminus managed to exit the ER and was tightly attached to widely-distributed Side-Cherry particles in the cytoplasm of the muscle fiber. The intracellular domain is therefore not required for the interaction with Side. Beat lacking the first Ig domain, however, did not co-localize with Side. The same was true for SideΔIg1-Cherry, which showed clearly diminished activity in preventing nuclear localization of Beat-GFP and in attracting motor axons. Thus, mutual recognition of Beat and Side *via* their first Ig domains is probably a key-regulatory step for their functional interaction.

Interestingly, full-length Beat localized to different subcellular compartments, depending on GFP being attached to either the N- or C-terminus. Expression of GFP-Beat-Cherry in both motor neurons and muscles demonstrated that GFP segregated from Cherry, indicating that Beat might be proteolytically cleaved in both tissues. Based on a band at ∼65 kDa recognized by anti-GFP antibodies in Western blots (Suppl. Fig. S1B), cleavage of GFP-Beat-Cherry might occur in or near the potential transmembrane region. Replacing this domain in BeatnewTM-GFP with a fairly well characterized transmembrane segment from human CD8, indeed prevented nuclear accumulation of Beat-GFP in muscles. Instead, BeatnewTM-GFP accumulated in ER compartments. Once this segment was removed, in BeatΔTM-GFP, ER exit was guaranteed and GFP was now clearly exposed on extracellular muscle surfaces. Since both constructs were also non-functional in rescue experiments in neurons, posttranslational processing appears to be an essential requirement for Beat function and requires the endogenous sequences at or near the potential transmembrane region. Otherwise, enrichment of Beat-GFP in muscle nuclei seems to be independent of an interaction with Side. In the absence of either the first or both extracellular Ig domains (BeatΔIg1-GFP, BeatCys-GFP), GFP still enriched in nuclei. Similarly, nuclear accumulations occurred in *side* mutant muscles (data not shown). Thus, nuclear import required proper processing in the ER and most of the Cys-rich C-terminal domain.

Our structure-function analysis also provides information on the orientation of Beat with respect to cellular membranes. Immunohistochemical stainings under non-permeabilizing conditions showed that the N-terminus is exposed on the muscle surface. This was independently confirmed by the deGradFP system (Caussinus et al., 2011). When GFP was in extracellular compartments, like in GFP-Beat-Cherry, the system did not exert any visible effects on GFP, neither its expression level nor its subcellular distribution. In contrast, fusion proteins exposing GFP to the cytoplasm were either re-localized or downregulated.

Based on these experiments, we propose a model for the membrane topology of Beat. Beat is most likely synthesized as a type-I transmembrane protein, with its N-terminus in the ER and its C-terminus in the cytoplasm but then gets proteolytically processed overtime. The N-terminal part remains surface-associated, whereas the C-terminal part translocates into the nucleus. Although most of our experiments have been performed in muscles, N- and C-terminal separation did occur in motor neurons, too. Sequences in or near the potential transmembrane region of Beat seem to be necessary for these sorting and maturation processes. In fact, modelling protein sequences predict exposure of a potential GPI-anchor signal sequence upon cleavage. Future experiments will hopefully uncover the molecular basis of these posttranslational modifications and their relevance for motor axon guidance.

## Data Availability

The original contributions presented in the study are included in the article/Supplementary Material, further inquiries can be directed to the corresponding author.

## References

[B1] Aberle H. Haghighi A. P. Fetter R. D. Mccabe B. D. Magalhaes T. R. Goodman C. S. (2002). Wishful thinking encodes a BMP type II receptor that regulates synaptic growth in Drosophila. Neuron 33, 545–558. 10.1016/s0896-6273(02)00589-5 11856529

[B2] Bazan J. F. Goodman C. S. (1997). Modular structure of the Drosophila Beat protein. Curr. Biol. 7, R338–R339. 10.1016/s0960-9822(06)00168-0 9197253

[B3] Bieber A. J. Snow P. M. Hortsch M. Patel N. H. Jacobs J. R. Traquina Z. R. (1989). Drosophila neuroglian: A member of the immunoglobulin superfamily with extensive homology to the vertebrate neural adhesion molecule L1. Cell 59, 447–460. 10.1016/0092-8674(89)90029-9 2805067

[B4] Bischof J. Maeda R. K. Hediger M. Karch F. Basler K. (2007). An optimized transgenesis system for Drosophila using germ-line-specific phiC31 integrases. Proc. Natl. Acad. Sci. U. S. A. 104, 3312–3317. 10.1073/pnas.0611511104 17360644 PMC1805588

[B5] Buch S. Melcher C. Bauer M. Katzenberger J. Pankratz M. J. (2008). Opposing effects of dietary protein and sugar regulate a transcriptional target of Drosophila insulin-like peptide signaling. Cell Metab. 7, 321–332. 10.1016/j.cmet.2008.02.012 18396138

[B6] Caussinus E. Kanca O. Affolter M. (2011). Fluorescent fusion protein knockout mediated by anti-GFP nanobody. Nat. Struct. Mol. Biol. 19, 117–121. 10.1038/nsmb.2180 22157958

[B7] Dickson B. J. (2002). Molecular mechanisms of axon guidance. Science 298, 1959–1964. 10.1126/science.1072165 12471249

[B8] Fambrough D. Goodman C. S. (1996). The Drosophila beaten path gene encodes a novel secreted protein that regulates defasciculation at motor axon choice points. Cell 87, 1049–1058. 10.1016/s0092-8674(00)81799-7 8978609

[B9] Holmes A. L. Heilig J. S. (1999). Fasciclin II and Beaten path modulate intercellular adhesion in Drosophila larval visual organ development. Development 126, 261–272. 10.1242/dev.126.2.261 9847240

[B10] Huelsmann S. Hepper C. Marchese D. Knoll C. Reuter R. (2006). The PDZ-GEF dizzy regulates cell shape of migrating macrophages via Rap1 and integrins in the Drosophila embryo. Development 133, 2915–2924. 10.1242/dev.02449 16818452

[B11] Kinold J. C. Pfarr C. Aberle H. (2018). Sidestep-induced neuromuscular miswiring causes severe locomotion defects in Drosophila larvae. Development 145, dev163279. 10.1242/dev.163279 30166331

[B12] Kolodkin A. L. Tessier-Lavigne M. (2011). Mechanisms and molecules of neuronal wiring: A primer. Cold Spring Harb. Perspect. Biol. 3, a001727. 10.1101/cshperspect.a001727 21123392 PMC3098670

[B13] Li H. Watson A. Olechwier A. Anaya M. Sorooshyari S. K. Harnett D. P. (2017). Deconstruction of the Beaten path-Sidestep interaction network provides insights into neuromuscular system development. Elife 6, e28111. 10.7554/eLife.28111 28829740 PMC5578738

[B14] Lin D. M. Goodman C. S. (1994). Ectopic and increased expression of Fasciclin II alters motoneuron growth cone guidance. Neuron 13, 507–523. 10.1016/0896-6273(94)90022-1 7917288

[B15] Mahr A. Aberle H. (2006). The expression pattern of the Drosophila vesicular glutamate transporter: A marker protein for motoneurons and glutamatergic centers in the brain. Gene Expr. Patterns 6, 299–309. 10.1016/j.modgep.2005.07.006 16378756

[B16] Mushegian A. R. (1997). The Drosophila Beat protein is related to adhesion proteins that contain immunoglobulin domains. Curr. Biol. 7, R336–R338. 10.1016/s0960-9822(06)00167-9 9197251

[B17] Ozkan E. Carrillo R. A. Eastman C. L. Weiszmann R. Waghray D. Johnson K. G. (2013). An extracellular interactome of immunoglobulin and LRR proteins reveals receptor-ligand networks. Cell 154, 228–239. 10.1016/j.cell.2013.06.006 23827685 PMC3756661

[B18] Pipes G. C. Lin Q. Riley S. E. Goodman C. S. (2001). The beat generation: A multigene family encoding IgSF proteins related to the beat axon guidance molecule in Drosophila. Development 128, 4545–4552. 10.1242/dev.128.22.4545 11714679

[B19] Ranganayakulu G. Schulz R. A. Olson E. N. (1996). Wingless signaling induces nautilus expression in the ventral mesoderm of the Drosophila embryo. Dev. Biol. 176, 143–148. 10.1006/dbio.1996.9987 8654890

[B20] Schindelin J. Arganda-Carreras I. Frise E. Kaynig V. Longair M. Pietzsch T. (2012). Fiji: An open-source platform for biological-image analysis. Nat. Methods 9, 676–682. 10.1038/nmeth.2019 22743772 PMC3855844

[B21] Siebert M. Banovic D. Goellner B. Aberle H. (2009). Drosophila motor axons recognize and follow a Sidestep-labeled substrate pathway to reach their target fields. Genes Dev. 23, 1052–1062. 10.1101/gad.520509 19369411 PMC2682951

[B22] Sink H. Rehm E. J. Richstone L. Bulls Y. M. Goodman C. S. (2001). Sidestep encodes a target-derived attractant essential for motor axon guidance in Drosophila. Cell 105, 57–67. 10.1016/s0092-8674(01)00296-3 11301002

[B23] Tan L. Zhang K. X. Pecot M. Y. Nagarkar-Jaiswal S. Lee P. T. Takemura S. Y. (2015). Ig superfamily ligand and receptor pairs expressed in synaptic partners in Drosophila. Cell 163, 1756–1769. 10.1016/j.cell.2015.11.021 26687360 PMC4804707

[B24] Tessier-Lavigne M. Goodman C. S. (1996). The molecular biology of axon guidance. Science 274, 1123–1133. 10.1126/science.274.5290.1123 8895455

[B25] Van Vactor D. Sink H. Fambrough D. Tsoo R. Goodman C. S. (1993). Genes that control neuromuscular specificity in Drosophila. Cell 73, 1137–1153. 10.1016/0092-8674(93)90643-5 8513498

[B26] Vogel C. Teichmann S. A. Chothia C. (2003). The immunoglobulin superfamily in *Drosophila melanogaster* and *Caenorhabditis elegans* and the evolution of complexity. Development 130, 6317–6328. 10.1242/dev.00848 14623821

[B27] Zito K. Parnas D. Fetter R. D. Isacoff E. Y. Goodman C. S. (1999). Watching a synapse grow: Noninvasive confocal imaging of synaptic growth in Drosophila. Neuron 22, 719–729. 10.1016/s0896-6273(00)80731-x 10230792

