# Supplementary material for: Molecular insights into the axon guidance molecules Sidestep and Beaten path
Source: Front Physiol. 2022 Nov 28;13:1057413. doi: 10.3389/fphys.2022.1057413 (PMC9743010; doi:10.3389/fphys.2022.1057413)
Supplement: Supplementary file 1 [file DataSheet1.pdf]

Supplementary Material

to

Molecular insights into the axon guidance molecules Sidestep and Beaten path

Caroline Heymann[1], Christine Paul[1,#], Na Huang[1,#], Jaqueline C. Kinold[1,#], Ann-Christin Dietrich[2,3] and Hermann Aberle[1,*]

[1] Heinrich Heine University Düsseldorf, Department of Biology, Institute for Functional Cell Morphology, Building 26-44-00, Universitätsstrasse 1, 40225 Düsseldorf, Germany

[2] University of Münster, Institute of Neuro- and Behavioral Biology, Badestrasse 1, 48149 Münster, Germany

[3] present address: University of Münster, Cells in Motion Interfaculty Centre, Waldeyerstraße 15, 48149 Münster, Germany

# authors contributed equally

Supplementary Figure S1

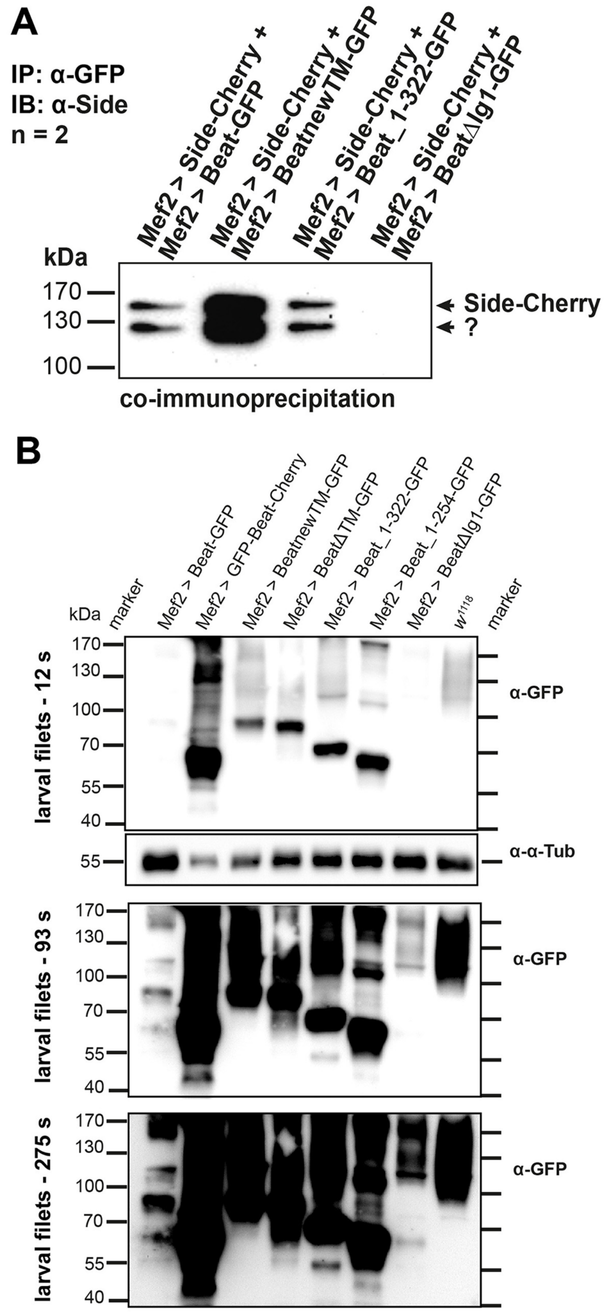

BeatΔIg1-GFP fails to co-immunoprecipitate with Side-Cherry.

(**A**) Western blot of anti-GFP immunoprecipitates developed with anti-Side antibodies. For each lane, the indicated GFP-tagged Beat constructs were expressed in body wall muscles. The resulting lysates were combined with body wall lysates of Side-Cherry-expressing larvae. Beat proteins were immunoprecipitated using anti-GFP antibodies, separated by SDS-PAGE and transferred to PVDF

membranes. Blots were probed with anti-Side antibodies. Beat-GFP, BeatnewTM-GFP and Beat_1-322-GFP but not BeatΔIg1-GFP interacted directly or indirectly with Side-Cherry. Side-Cherry migrates at 150 kDa, whereas the lower band is believed to represent a degradation product. n = 2 independent experiments.

(**B**) Expression levels of various Beat proteins fused to GFP in larval muscles. Western blot of lysates from body wall preparations of dissected w^{1118} larvae expressing the indicated Beat constructs in muscles using Mef2-Gal4. The blot was developed with anti-GFP antibodies (12s exposure time). GFP-Beat-Cherry was expressed at very high levels and showed a prominent band at 65 kDa. The remaining four fusion proteins ran at the expected sizes. Beat-GFP and BeatΔIg1-GFP were notoriously difficult to detect in Western blots in our hands but were detectable at longer exposures (93s and 275s). w^{1118} larvae showed some background signals. The same membrane was re-probed with anti-α-Tubulin antibodies as a loading control (20s exposure time). n = 3 independent experiments.

Supplementary Figure S2

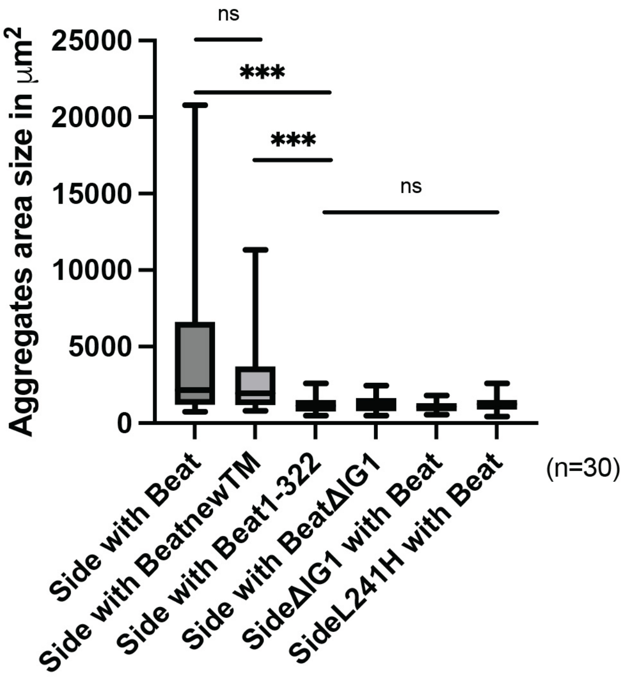

Surface areas of cell aggregates formed in S2 cell-cell interactions assays.

S2 cells were transfected with the indicated Cherry-tagged Side constructs and then mixed with S2 cells expressing the indicated GFP-tagged Beat constructs. Mixed cell suspensions were incubated on

a rocking platform to allow for cell-cell interactions. Side-Cherry induced large aggregates with Beat-GFP and BeatnewTM transfected cells but not with Beat_1-322-GFP transfected cells. The surface area of randomly selected aggregates was determined using Fiji is just ImageJ software. Two-tailed Mann-Whitney U test, n = 30, ns, non-significant, *** p≤0.001.

Supplementary Figure S3

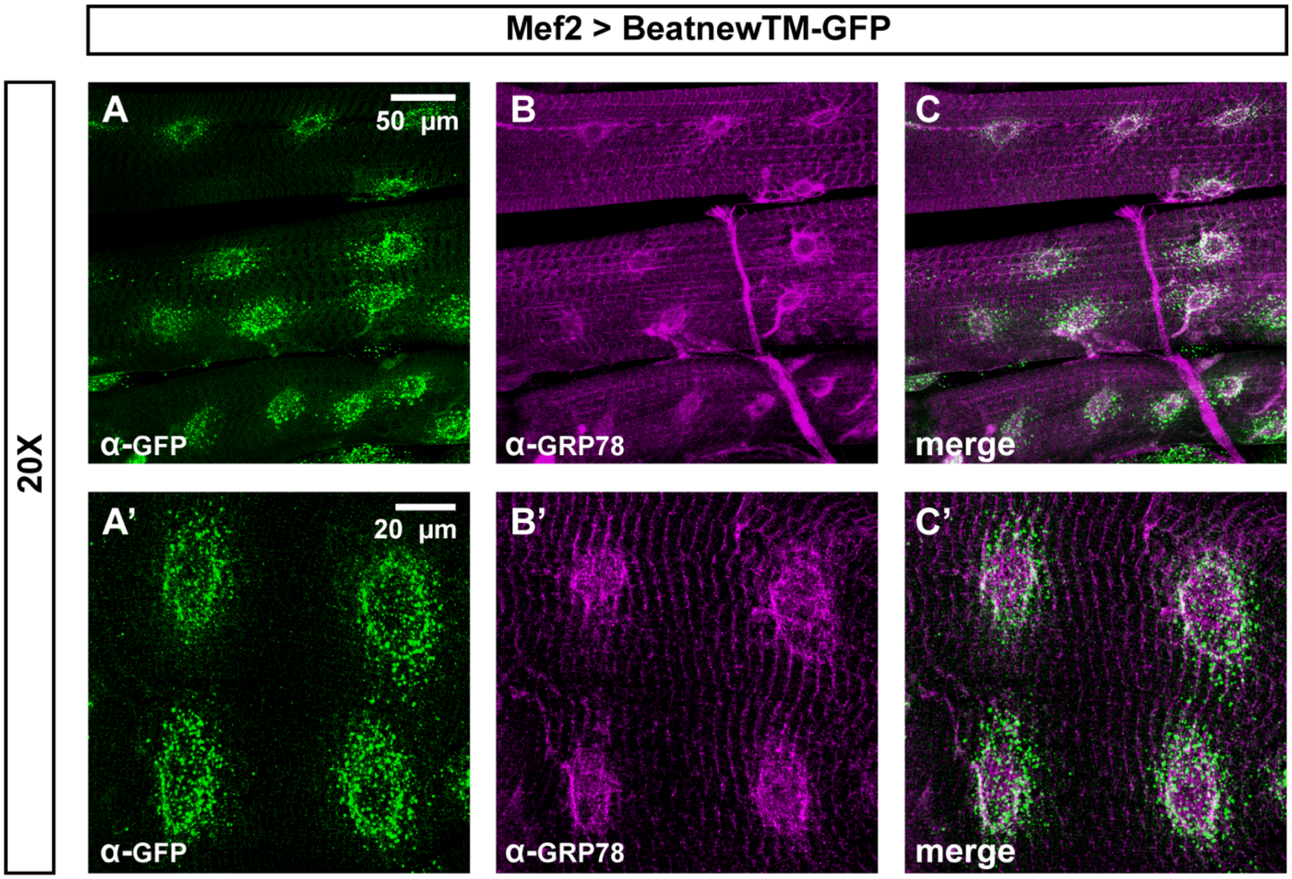

BeatnewTM-GFP co-localizes with the ER marker GRP78 (also called BIP, Binding Immunoglobulin Protein) in muscles.

(**A-C**) Confocal images of dissected third instar larvae expressing BeatnewTM-GFP under control of Mef2-Gal4. (**A**) Anti-GFP antibodies (green) reveal that BeatnewTM-GFP localizes to a restricted subcellular compartment surrounding nuclei. (**B**) ER compartments are marked with anti-GRP78 (magenta). (**C**) Merged images. (**A'-C'**) Magnified views of a similar region showing that BeatnewTM-GFP largely overlaps with GRP78 signals.

Scale bars: 50 and 20 µm.

Supplementary Figure S4

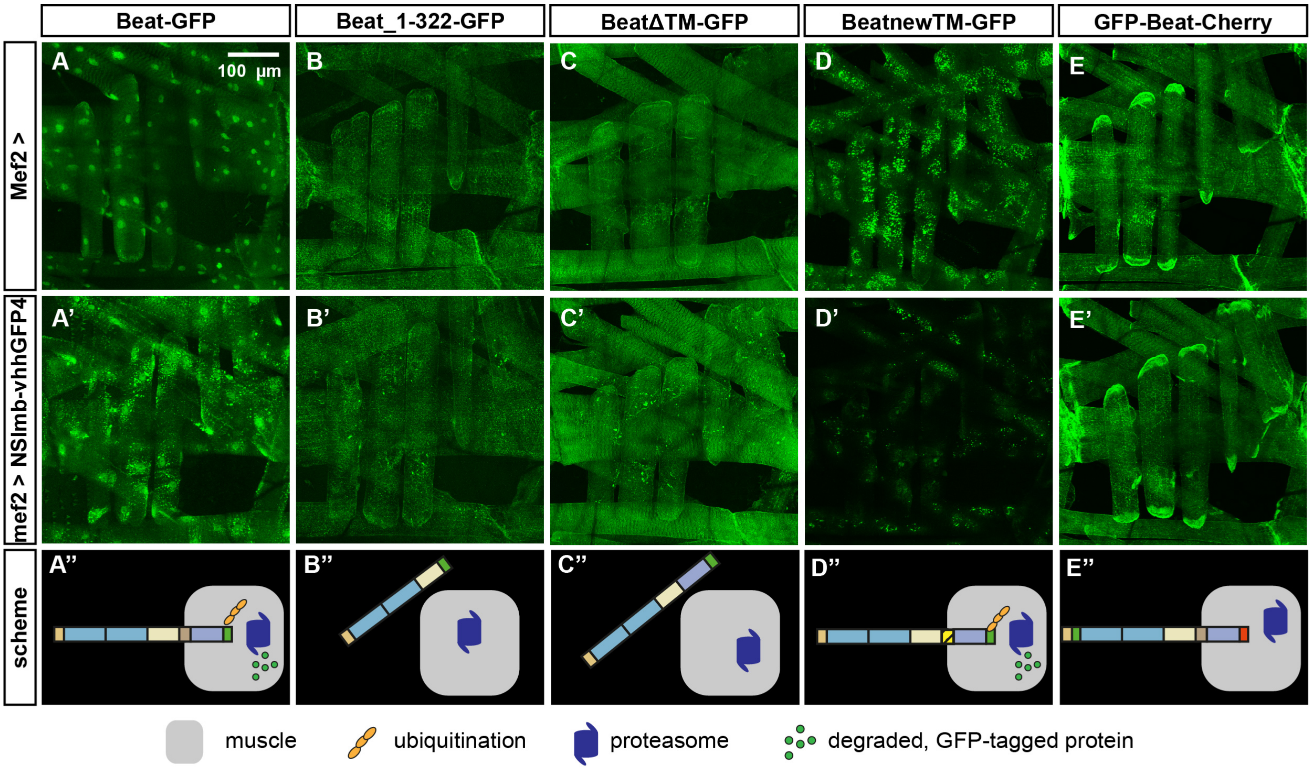

Suppl. Fig. S4

Beat extracellular GFP-tags escape degradation or re-localization by the deGradFP system.

(**A-E**) Confocal micrographs acquired through the translucent cuticle of intact third instar larvae overexpressing the indicated Beat constructs in muscles (Mef2-Gal4). (**A**) Beat-GFP localizes to muscle nuclei. (**B-C**) Beat_1-322-GFP and BeatΔTM-GFP, both lacking the potential transmembrane region, distribute over the entire muscle surface. (**D**) BeatnewTM-GFP is withheld in ER compartments. (**E**) GFP-Beat-Cherry, carrying GFP at the N-terminus, is targeted to the muscle membrane.

(**A'-E'**) Co-expression of NSlmb-vhhGFP4 (GFP-binding nanobody recruiting E3-ubiquitin ligases) and the indicated Beat constructs in muscles. (**A'**) Interaction with the nanobody traps Beat-GFP in the ER and prevents its transport to nuclei but it is not degraded. (**B'-C'**) Both Beat_1-322-GFP and BeatΔTM-GFP are not affected by the nanobody, as the GFP tag is likely hidden in extracellular compartments. (**D'**) BeatnewTM-GFP is strongly downregulated. (**E'**) Expression levels and localization of GFP-Beat-Cherry is not affected as GFP is not exposed in the cytoplasm.

(**A''-E''**) Schematic models of the effects of the deGradFP system on various Beat constructs. Only fusion proteins with cytoplasmic GFP-tags are ubiquitinated (orange ellipses) and degraded or re-localized (green circles). Scale bar: 100 μm.

Supplementary Figure S5

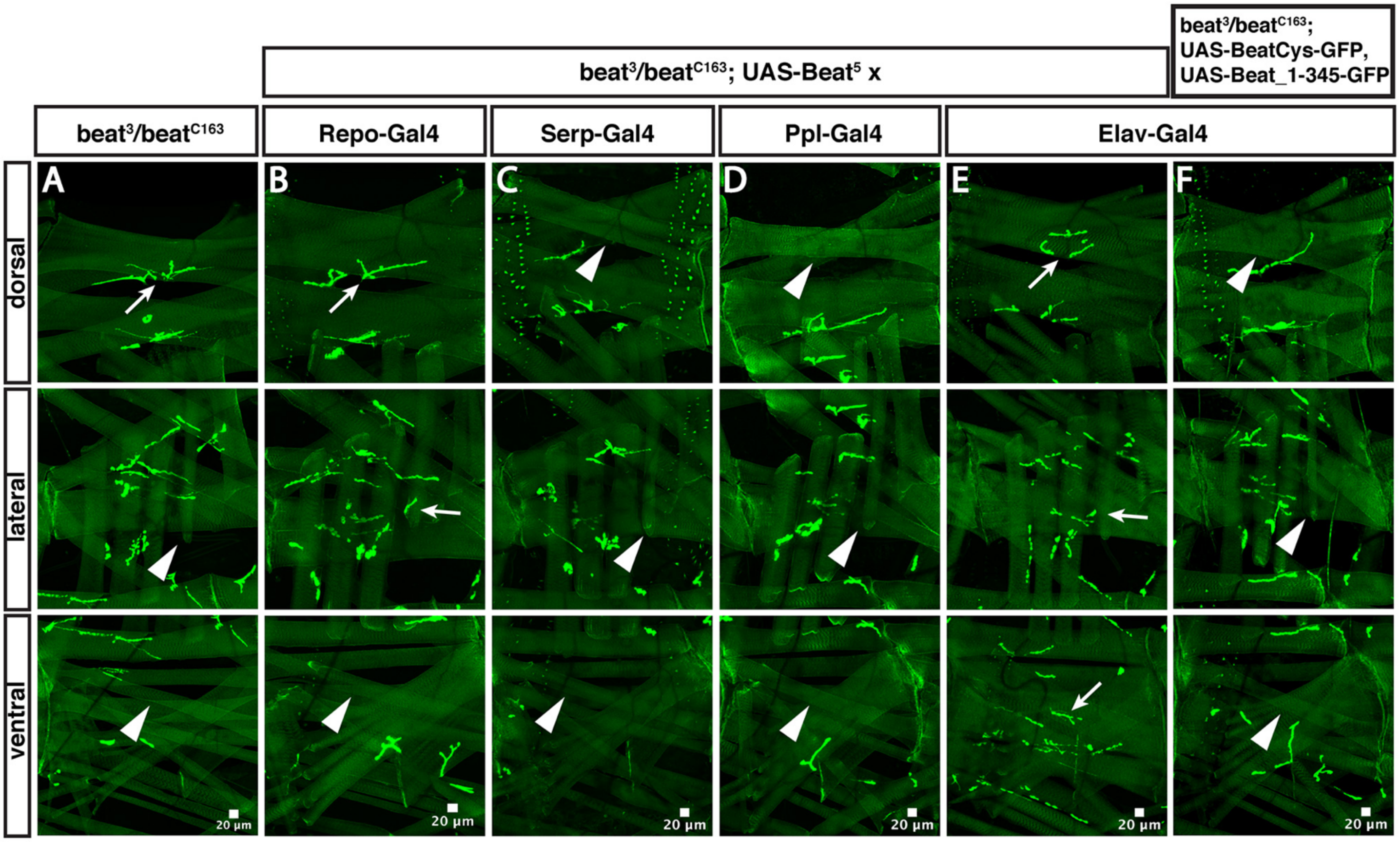

Expression of a functional *beat* cDNA in non-neuronal tissues does not rescue *beat* mutant innervation phenotypes.

(**A-F**) Confocal images acquired through the transparent cuticle of third instar larvae showing dorsal, lateral and ventral body wall regions of the same abdominal hemisegment. Muscles and NMJs are stained with ShGFP (green), which is expressed in all genetic backgrounds. While expression of a full-length *beat* cDNA in glia (**B**, Repo-Gal4), hemocytes (**C**, Serpent-Gal4) or fat body (**D**, Pumpless-Gal4) did not rescue *beat* mutant innervation phenotypes, expression in all neurons (Elav-Gal4) fully

restored the innervation pattern (**E**). Splitting Beat in half by co-expression of an N- and C-terminal fragment of Beat in all neurons, however, did not rescue (**F**). Arrows mark correctly innervated muscles M9 (dorsal), M24 (lateral), M14 (ventral). Arrowheads mark the respective non-innervated fibers. Other muscles lack NMJs as well but are not labelled. Scale bars: 20 µm.

Supplementary Figure S6

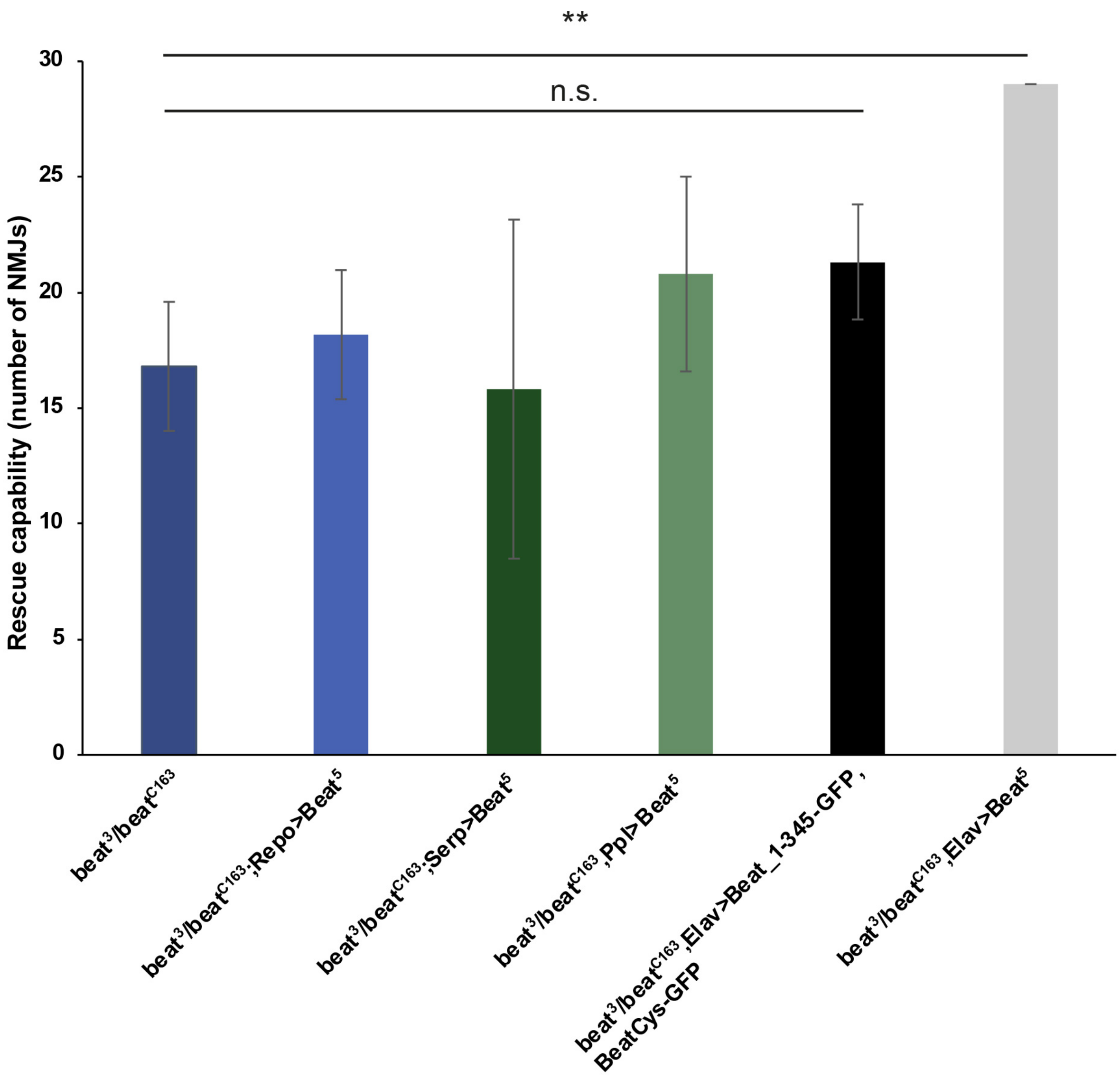

Quantification of the rescue ability of untagged Beat in various tissues.

A wild-type Beat cDNA was expressed in various tissues in a *beat* mutant background and 29 out of 30 NMJs stained with ShGFP were evaluated per hemisegment (NMJ on muscle 25 was not evaluated). While *beat* mutant larvae display approximately 17 NMJs per hemisegment, ectopic expression of Beat in glia (Repo-Gal4), hemocytes (Serpent-Gal4) or fat body (Pumpless-Gal4) did not or only insignificantly improve the degree of innervation (16-20 NMJs). Providing Beat in postmitotic neurons using Elav-Gal4, however, completely rescued the phenotype back to wild-type levels (29 NMJs). Simultaneous expression of a N- and C-terminal fragment, however, was not sufficient to restore a wild-type innervation pattern (21 NMJs). Different colors represent individual genotypes. n = 5-6 hemisegments per genotype, n.s., not significant, ** ≤ 0.01.
